# Variations in Grain Traits among Local Rice Varieties Collected More Than Half-Century Ago in Indochinese Countries

**DOI:** 10.3390/plants12010133

**Published:** 2022-12-27

**Authors:** Sathya Lim, Anna Onoda, Chhourn Orn, Hiromu Iwamoto, Ryo Ishikawa, Hiroki Saito, Yutaka Sato, Takashige Ishii

**Affiliations:** 1 Graduate School of Agricultural Science, Kobe University, 1-1 Rokkodai, Nada-ku, Kobe 657-8501, Hyogo, Japan; 2 Cambodian Agricultural Research and Development Institute (CARDI), Phnom Penh P.O. Box 01, Cambodia; 3 Tropical Agriculture Research Front, Japan International Research Center of Agricultural Science, Ishigaki 907-0002, Okinawa, Japan; 4 Department of Genomics and Evolutionary Biology, National Institute of Genetics, Mishima 411-8540, Shizuoka, Japan

**Keywords:** rice, local varieties, grain size, Indochinese countries, genetic resources

## Abstract

More than half-century ago, local rice varieties were collected from Indochinese countries (Cambodia, Thailand, Laos, and Vietnam). Of these, 162 local varieties were examined for 7 grain-size traits: seed length/width/thickness, brown rice length/width/thickness, and 100-seed weight. Since these traits varied considerably, a survey of functional mutations was performed in the genes related to these traits. In total, 19 markers (12 InDel and 7 dCAPS markers) were used to investigate the mutations at 14 grain-size loci of *GW2*, *GS2*, *qLGY3*, *GS3*, *GL3.1*, *TGW3*, *GS5*, *GW5*, *GS6*, *TGW6*, *GW6a*, *GLW7*, *GL7*, and *GW8.* Significant allele effects were observed with six markers detecting base substitution mutations at *GW2* and *GS3* and insertion/deletion mutations at *GS5*, *GW5*, and *GW6a*, suggesting that these mutations might have affected the grain trait and caused variation among local varieties in the Indochinese countries. In addition to grain size, the hull color, grain color, and glutinosity were also examined using a survey of loss-of-function mutations at major responsible loci. Most phenotypes were reflected based on functional mutations at these loci. Since the local varieties have wide genetic variation, they are important genetic resources for future rice breeding.

## 1. Introduction

In the mid-1950s, the Japanese Society of Ethnology planned to have a full-scale academic expedition named the “Southeast Asian Rice Farming Ethnic Culture Research Mission” [1]. The first research team was comprised of 18 experts in linguistics, ethnology, archeology, and other research fields. They were dispatched to Cambodia, Thailand, Laos, and Vietnam between 1957 and 1958. One of the participants in this expedition was the late Professor Hideo Hamada, an expert in agriculture from the Hyogo University of Agriculture, Japan. He conducted agricultural field surveys in these four countries and collected 1208 accessions of local rice varieties in 1957 and 1958 [2]. They are valuable genetic resources because they were collected before the major social disorders in Indochinese countries, such as the Vietnam War in the 1960s and the Cambodian Civil War in the 1970s, and before the advent of the green revolution in the 1960s, which led to the development of a number of modern rice cultivars that were introduced to these countries. Additionally, these important local varieties supported traditional rice farming in Indochinese countries. Prof. Hamada donated the seed specimens and related materials to the Faculty of Agriculture, Kobe University, the institution that was the successor of Hyogo University of Agriculture. The seeds were stored for a long time at room temperature and have lost their germination ability.

Recently, we came to know that part of the seed collections had also been transferred to the National Institute of Genetics, Japan, in 1959. A total of 638 accessions were registered as genetic resources of cultivated rice in Oryzabase (https://shigen.nig.ac.jp/rice/oryzabase/) accessed on 22 January 2020. However, they have rarely been used for genetic studies because the data in their collection are not precise and seed distribution was limited. Of these, we could multiply the seeds of 162 local varieties. In general, local varieties have more genetic variations than do the modern cultivars because the local varieties adapted to the various local conditions, and they were not subjected to strict selection in breeding program. Therefore, we expect to observe varied seed characters among the 162 local varieties from the Indochinese countries.

In rice, several quantitative trait loci (QTLs) for grain size and the corresponding genes have been investigated. Currently, more than 10 QTLs have been functionally identified and the causal mutations have been estimated: *GW2* [3], *GS2* [4], *qLGY3* [5], *GS3* [6], *GL3.1* [7], *TGW3* [8], *GS5* [9], *GW5* [10,11], *GS6* [12], *TGW6* [13], *GW6a* [14], *GLW7* [15], *GL7* [16], and *GW8* [17]. Previously, PCR-based markers were developed to detect functional mutations at these gene loci [18]. These markers are useful for surveying the genotypes of putative functional mutations. In addition to grain size, the hull color, grain color, and glutinosity can be easily examined. These are qualitative traits controlled by dominant genes: black hull color by *Bh4*, red pericarp color by *Rc*, and non-glutinosity by *Waxy*. Loss-of-function mutations in these genes leads to the formation of straw hulls [19], white pericarps [20], and glutinosity [21], respectively.

In this study, we first examined grain size, hull color, grain color, and glutinosity using 162 local varieties collected more than 60 years ago in Indochinese countries. PCR-based markers were further used to survey the functional mutations of the responsible genes. By comparing morphological and genotypic data, we attempted to reveal the genetic mechanisms on grain traits among the local varieties that support traditional rice farming in the Indochinese countries.

## 2. Results

### 2.1. Verification of Collection Passport Data on Local Varieties

Parts of the local varieties collected by Prof. Hamada were maintained at the National Institute of Genetics, Japan. Of these, the seeds of the 162 local varieties (49 Cambodian, 30 Thai, 20 Laotian, and 63 Vietnamese varieties) were multiplied. Since the precise collection information had not been transferred to the National Institute of Genetics, their passport data were verified based on the original collection-related objects such as the seed envelopes (Figure S1), trip records, and trip reports [1,2] kept at Kobe University. Thus, the names and sources of 107 varieties (66.0%) were corrected. The passport data with additional information, such as local classifications [2], are presented in Table S1.

### 2.2. Grain-Size Variation among Local Varieties

**Figure 1:**
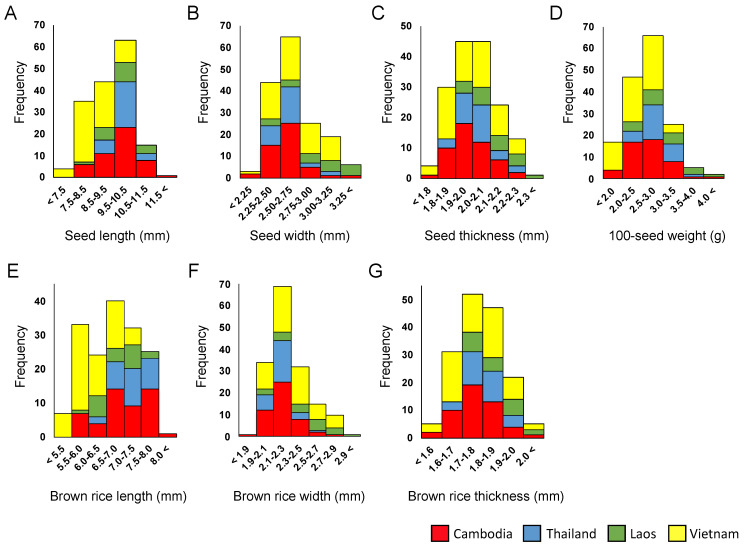
Frequency distributions of grain traits observed among 162 local rice varieties in four Indochinese countries. (**A**) Seed length. (**B**) Seed width. (**C**) Seed thickness. (**D**) 100-seed weight. (**E**) Brown rice length. (**F**) Brown rice width. (**G**) Brown rice thickness. Column charts are colored in red, blue, green, and yellow for Cambodian, Thai, Laotian, and Vietnamese varieties, respectively.

**Figure 2:**
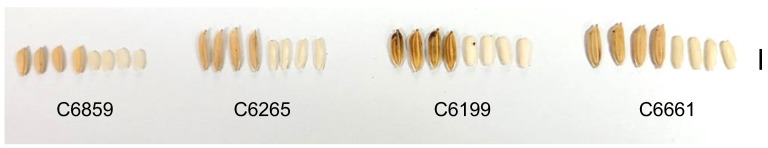
Hulled and dehulled seeds of four local rice varieties. The shortest and the longest seed lengths among the 162 local varieties were observed with C6859 and C6661, respectively. The narrowest and the widest seed widths were observed with C6265 and C6199, respectively. The accession C6661 showed the highest value also for 100-seed weight. The accession C6199 is a glutinous variety with brown furrowed hulls. Scale bar = 5 mm.

In this study, the grain size was examined using seeds (with hull) and brown rice (without hull). In total, 7 grain traits were measured among the 162 local varieties: seed length, seed width, seed thickness, brown rice length, brown rice width, brown rice thickness, and 100-seed weight (Table S2). Continuous distributions were observed for all seven traits (Figure 1); the ranges and averages of these traits are summarized in Table 1. The shortest (7.15 mm) and longest (11.52 mm) seed lengths were observed in the Vietnamese accession C6859 and the Cambodian accession C6661, respectively (Figure 2). In contrast, C6265 (Cambodia) and C6199 (Laos) had the narrowest (2.20 mm) and widest (3.46 mm) seed widths, respectively (Figure 2). Seed thickness values ranged from 1.75 to 2.30 mm, with the average of 2.01 mm. Regarding 100-seed weight, the lowest (1.71 g) and highest values (4.40 g) were observed for C7042 (Vietnam) and C6661 (Cambodia) accessions, respectively. As for the brown rice, the length, width, and thickness were highly correlated with seed size, and their correlation coefficient values were greater than 0.965 (Table 2). In general, considerable variation in grain size was detected among the local varieties collected approximately 60 years ago from the Indochinese countries. At the country level, seed and brown rice lengths were predominantly long and short among the Thai and Vietnamese varieties, respectively (Table 1). However, seed and brown rice width and thickness values were higher among the Laotian varieties. These tendencies reflected the positive and negative correlations between seed and brown rice traits (Table 2). Regarding 100-seed weight, higher and lower averages were observed for the Laotian and Vietnamese varieties, respectively.

### 2.3. Detection of Functional Mutations on Seed Size Using PCR-Based Markers

Previously, PCR-based markers were developed to detect 20 functional mutations (12 length mutations, 7 base substitutions, and 1 duplication) at 14 grain-size loci: *GW2*, *GS2*, *qLGY3*, *GS3*, *GL3.1*, *TGW3*, *GS5*, *GW5*, *GS6*, *TGW6*, *GW6a*, *GLW7*, *GL7*, and *GW8* [18]. In this study, 19 markers (12 InDel and 7 dCAPS markers) were used to detect these mutations (Table S3). In accordance with a previous study, the markers were named after the combination of locus name and mutation type, i.e., GW2_SNP, GW2_InDel, GS2_SNP, qLGY3_InDel, GS3_SNP1, GS3_SNP2, GL3.1_SNP, TGW3_SNP, GS5_InDel, GW5_InDel, GS6_InDel, TGW6_InDel, TGW6_SNP, GW6a_InDel1, GW6a_InDel2, GLW7_InDel, GL7_InDel1, GL7_InDel2, and GW8_InDel. Most of the primers were redesigned, and mutations were surveyed based on the amplified product sizes, as shown in Table S4.

**Figure 3:**
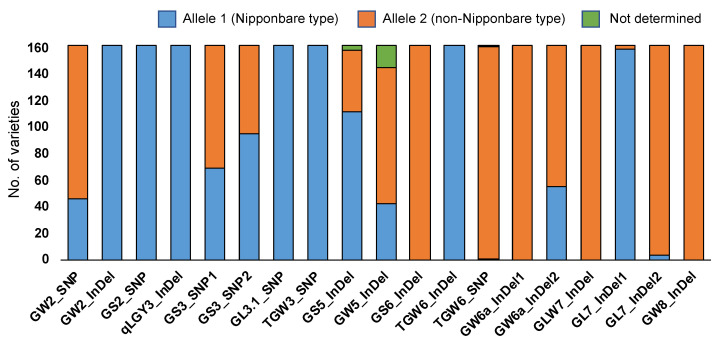
Allele frequencies revealed using 19 PCR-based markers at 14 grain-size loci observed among the 162 local varieties. Alleles 1 and 2 were assigned for the Nipponbare and non-Nipponbare types, respectively. Some varieties could not be determined with the allele types.

Using the 19 markers, the allele types (Allele 1, a reference variety of Nipponbare type; Allele 2, an alternative non-Nipponbare type) (Table S4) and their frequencies were examined among the 162 local varieties from the Indochinese countries (Figure S2, Table S5, Figure 3). Only reference alleles of the Nipponbare type were detected for six markers (GW2_InDel, GS2_SNP, qLGY3_InDel, GL3.1_SNP, TGW3_SNP, and TGW6_InDel), indicating that the other previously identified alleles were rare ones. In contrast, four markers (GS6_InDel, GW6a_InDel1, GLW7_InDel, and GW8_InDel) were monomorphic and alleles were of the non-Nipponbare type. For three markers (TGW6_SNP, GL7_InDel1, and GL7_InDel2), most varieties (more than 159 varieties) showed one allele type. The remaining six markers (GW2_SNP, GS3_SNP1, GS3_SNP2, GS5_InDel, GW5_InDel, and GS6a_InDel2) gave two allele groups consisting of more than 42 varieties. The average values of seven grain traits were calculated for two groups (Table 3), and the allele effects were estimated using Student’s *t*-test (Table 4). Significant differences between the groups were detected for all six markers; highly significant values at the 1% level were observed for seed length, brown rice grain length, and 100-seed weight. In most of these trait combinations, Allele 1 (Nipponbare-type allele) showed significant increase effects in trait values, except for GS3_SNP1. Regarding width and thickness, Allele 1 (Nipponbare-type allele) of GW5_InDel had a significant increase effect.

### 2.4. Evaluation of Other Seed Morphological Traits among Local Varieties

In addition to grain size, three morphological traits (hull color, pericarp color, and glutinosity) were examined at the same time (Table S6). Among the 162 local varieties, none of the varieties had black hulls, 43 showed intermediate types (with light brown color or brown furrows on a straw background) (Figure 2), and the remaining 119 had straw-colored hulls. In the dehulled condition, red, purple, and white, pericarps were observed in 3, 3, and 156 varieties, respectively. Regarding glutinosity, 25 had glutinous grains and 137 had normal grains (Figure 2).

In rice, the major loci for hull color, pericarp color, and glutinosity are *Bh4*, *Rc*, and *Waxy*, respectively. Their notable loss-of-function mutations were reported to be caused by a 22 bp deletion, 14 bp deletion, and 23 bp insertion in the coding regions, respectively (Table S7). Using PCR-based markers on these mutation sites, the length differences were surveyed in the 162 local varieties (Figure S2, Table S6). As a result, all varieties had loss-of-function mutations at *Bh4*, whereas 159 and 25 varieties showed mutations at *Rc* and *Waxy* loci, respectively.

## 3. Discussion

In this study, the passport data for 162 local varieties were first verified based on the data noted on the original packages used for collection. The revised passport data, with additional information on local classification, will be reflected in the germplasm database of the National Institute of Genetics (Table S1). They are quite important for utilizing the varieties and also for returning the germplasms to their original countries. In addition to the 162 local varieties, we retained the original collection-related packaging materials for the remaining accessions collected by Prof. Hamada. As they are also registered in the germplasm database, their passport data will be revised for future germplasm distribution.

Grain traits of the 162 local varieties from the Indochinese countries were examined, and considerable variation was observed in length, width, thickness, and weight (Figure 1, Table S2). These rice varieties had supported traditional rice farming; hence, these plants might have adapted to each unique local environment. Geographically, the varieties from Thailand tended to have long seeds and brown rice grains, whereas Vietnamese varieties had short seeds. Laotian varieties generally had wide and thick seeds/grains with a heavier seed weight. Probably, many upland rice varieties with large seeds/grains were included in the Laos collection.

Using 19 PCR-based markers, the functional mutations (12 length mutations, 7 base substitutions) at 14 grain-size loci of *GW2*, *GS2*, *qLGY3*, *GS3*, *GL3.1*, *TGW3*, *GS5*, *GW5*, *GS6*, *TGW6*, *GW6a*, *GLW7*, *GL7*, and *GW8* were surveyed (Table S5). Of these, 13 markers showed monomorphic or almost monomorphic allele types among the 162 local varieties (Figure 3). The remaining six markers (GW2_SNP, GS3_SNP1, GS3_SNP2, GS5_InDel, GW5_InDel, and GS6a_InDel2) yielded two allele groups consisting of more than 42 varieties. Student’s *t*-test was carried out with the averages between the two allele groups (Table 4). Allele 1 (Nipponbare-type allele) detected by GW5_InDel showed a highly significant increase in seed/grain width and thickness. Allele 2 had an approximately 1000–1200 bp insertion variation in the upstream region, which may change the expression level of the *GW5* gene. These results suggest that this mutation is one of the major factors that increases seed/grain width and thickness among local varieties in Indochinese countries.

Among the other five markers, significantly increased effects on seed/grain length and thickness and 100-seed weight were observed with Allele 1 of GW2_SNP, GS3_SNP2, GS5_InDel, and GS6a_InDel2, and Allele 2 of GS3_SNP1. In the cases of GS3_SNP1 and GS3_SNP2, the mutations were located at the same locus, *GS3*. Since most of the varieties (142 out of 162) had either mutation at *GS3* (Table S5), a tight linkage exists between the two markers. Previously, a base substitution in exon 2 at *GS3* (GS3_SNP1) was reported to result in an early stop codon and an increase in grain length [6], whereas the effect of the other SNP (GS3_SNP2) has not been clarified [18,22]. Therefore, we prioritized GS3_SNP1 as the causal mutation in *GS3*. To evaluate the gene pyramiding effects on seed/grain traits, five combinations of genotypes at four marker loci were extracted from the whole data (Table S8). A total of 12 varieties housed all 4 positive alleles with increasing effects, that is, Allele 1 of GW2_SNP, GS5_InDel, and GS6a_InDel2 and Allele 2 of GS3_SNP1 (hereafter “1112” allele combination in genotypic order of GW2_SNP, GS5_InDel, GS6a_InDel2, and GS3_SNP1). Their average values for seed/grain length and 100-seed weight were much higher than the overall averages, confirming the pyramiding effect of the alleles. Trait values of other four combinations with three increasing-effect alleles (“2111”, “1212”, “1122”, and “1111” allele combinations) were further examined and compared with those of “1112” having all positive alleles. Some combinations gave higher values for seed/grain thickness and 100-seed weight than “1112”, suggesting that the allele interaction is complex for these traits. As expected, similar results were also observed in the comparison of seed/grain widths, which were not related to the increasing effects of the alleles. In contrast, all four combinations gave lower values on seed/grain length than that of “1112”. Interestingly, the trait values of “2111” and “1111” dropped drastically, suggesting that Allele 1 of GW2_SNP and Allele 2 of GS3_SNP1 have strong positive effects on these traits. These results indicate that the four mutations might have affected seed/grain length variation among local varieties in Indochinese countries.

In addition to grain size, the hull color, grain color, and glutinosity were examined with a survey of loss-of-function mutations at major responsible loci. None of the varieties had black-colored hulls because all varieties housed non-functional alleles at the *Bh4* locus. Intermediate types were observed among the 43 varieties. They had hulls with a light brown color or brown furrows on a straw background, indicating that other genes may be responsible for the brown pigmentation. As for pericarp color, all varieties with white pericarps had non-functional alleles, and three varieties with red pericarps did not have the deletions. These pericarp phenotypes fit the genotypes at *Rc*. However, three varieties showed purple pericarps with non-functional alleles at *Rc*. The *Rc* gene is a positive regulator of the red pigment proanthocyanidin but not the purple pigment anthocyanin. Therefore, other genes, such as *Kala* genes [23], may be involved in the accumulation of purple pigment in pericarps. In general, glutinous rice is commonly found in Indochinese countries. Among the 162 varieties, 23 bp insertions were detected in 25 glutinous varieties but not in 137 normal varieties. The glutinosity of four varieties (C6655, C6666, C6697, and C6784) was inconsistent with the local rice classification (Table S1), indicating that the original varieties might have consisted of mixed lines. Since all the glutinous varieties (4 Cambodian, 2 Thailand, 14 Laotian, and 5 Vietnamese varieties) shared the same mutation at *Waxy* locus, glutinosity must have originated from a single mutation in Indochinese countries.

## 4. Materials and Methods

### 4.1. Plant Materials

A total of 162 local varieties from Indochinese countries (Cambodia, Vietnam, Laos, and Thailand) were used in this study. They were collected by Prof. Hamada of Hyogo University of Agriculture, Japan, in 1957 and 1958. The seeds were obtained from the National Institute of Genetics, Japan, and seed multiplication from single plants was carried out at Kobe University, Japan. The updated passport data with additional information (local rice classification) are listed in Table S1.

### 4.2. Grain Morphological Traits

The local varieties were grown and the seeds were harvested in the university field, Kobe University, Japan (34°43′35″ N, 135°14′7″ E). In total, seven quantitative traits were examined: seed length, seed width, seed thickness, brown rice length, brown rice width, brown rice thickness, and 100-seed weight. For grain size, 10 intact seeds or brown rice grains were measured for each variety using a digital Vernier caliper with data output function (CD-AX, Mitutoyo, Japan), the average values were calculated, and seed weight was checked with 100 seeds. In addition, three morphological traits (hull color, pericarp color, and glutinosity) were examined. These were qualitative traits and no segregation was found within the varieties. Hull colors were classified into three categories: black, intermediate (light brown or brown furrows on a straw background), and straw. The pericarp was clearly recognized with red, purple, and white colors. Glutinosity was judged based on the opacity of the grains (Figure 2).

### 4.3. Detection of Functional Mutations at Grain-Related Loci Using PCR-Based Markers

Previously, 20 functional mutations at 14 grain-size loci (*GW2*, *GS2*, *qLGY3*, *GS3*, *GL3.1*, *TGW3*, *GS5*, *GW5*, *GS6*, *TGW6*, *GW6a*, *GLW7*, *GL7*, and *GW8*) were examined using PCR-based markers [18]. Of these, 19 mutations were surveyed using 12 InDel and 7 dCAPS markers in this study (Table S4). Although most primers were redesigned for better amplification, the mutation codes of the markers were obtained from a previous study (Table S2).

PCR was performed in a 20 μL mixture containing 0.2 μM of each primer and 10 μL of 2× Quick Taq HS DyeMix (TOYOBO); total DNA extracted from young leaves was used as a template. For the markers of GS3_SNP1 and GW8_InDel, amplification was carried out in a 20 μL mixture containing 0.5 μM of each primer, 200 μM dNTP, 10 μL of 2× PCR buffer, and 0.1 U of KOD FX Neo polymerase (TOYOBO). The amplification conditions were as follows: initial denaturation at 94 °C for 5 min, followed by 35 cycles of 30 s at 94 °C, 30 s at 55 °C, 1 min at 72 °C, and a final extension for 5 min at 72 °C. The annealing temperature for the GS2_SNP marker was set to 65 °C instead of 55 °C, and the extension step for the GW5_InDel marker was changed from 1 min at 72 °C to 4 min at 72 °C. In the case of dCAPS markers, enzyme digestion was performed in a 15 μL mixture containing 5 μL of each amplified product, 1.5 μL of 10× Buffer, and 2 U of enzyme.

The DNA fragments were electrophoresed on a 4% polyacrylamide gel or 1.5% agarose gel (only for the GW5_InDel marker), and the band patterns were visualized using silver or fluorescent staining methods, respectively [24]. Allele scoring was performed based on the fragment sizes: Allele 1 represents a reference allele based on the Nipponbare sequences, and Allele 2 is an alternative allele (non-Nipponbare type) (Table S4).

### 4.4. Allele Genotyping at Major Loci for Hull Color, Pericarp Color, and Glutinosity

Regarding hull color, pericarp color, and glutinosity, major loss-of-function mutations have been reported to be caused by the 22 bp deletion at *Bh4*, 14 bp deletion at *Rc*, and 23 bp insertion at *Waxy* loci [19,20,21]. Primer pairs were designed to detect differences in length (Table S7). They were subjected to PCR in a 20 μL mixture containing 0.2 μM of each primer and 10 μL of 2× Quick Taq HS DyeMix (TOYOBO). Amplification was carried out as follows: initial denaturation at 94 °C for 5 min, followed by 35 cycles of 30 s at 94 °C, 30 s at 55 °C, 1 min at 72 °C, and a final extension for 5 min at 72 °C. The PCR products were electrophoresed on a 4% polyacrylamide gel, and band patterns were visualized using the silver staining method [24]. Based on the band sizes, functional and non-functional alleles were estimated at the loci for hull color, pericarp color, and glutinosity.

## 5. Conclusions

More than 60 years ago, before the occurrence of the major social disorder events in Indochinese countries and the release of modern breeding varieties, seeds of local rice varieties were collected from four Indochinese countries (Cambodia, Thailand, Laos, and Vietnam). In this study, 162 of those local varieties were examined for grain traits, and a survey of functional mutations in the related genes was performed. Considerable variation was detected in grain traits with responsible mutations, implying high genetic variations also exist in other traits. We believe that the local varieties analyzed here are important genetic resources that will provide useful traits for future rice breeding programs.

## Figures and Tables

**Table 1:** Ranges and averages of seven grain traits observed among 162 local rice varieties.

Trait ^a^	Min.	Max.	Ave.	Ave.			
				Cambodia	Thailand	Laos	Vietnam
SL (mm)	7.15	11.52	9.32	9.69	9.93	9.72	8.62
SW (mm)	2.20	3.46	2.68	2.58	2.58	2.97	2.71
ST (mm)	1.75	2.30	2.01	1.99	2.03	2.11	1.99
BL (mm)	5.14	8.27	6.69	6.99	7.23	6.83	6.15
BW (mm)	1.77	2.90	2.27	2.19	2.18	2.46	2.32
BT (mm)	1.54	2.04	1.79	1.77	1.81	1.87	1.78
100W (g)	1.71	4.40	2.61	2.62	2.82	2.98	2.39

^a^ SL: seed length. SW: seed width. ST: seed thickness. BL: brown rice length. BW: brown rice width. BT: brown rice thickness. 100W: 100-seed weight.

**Table 2:** Correlation coefficient values between seven grain traits observed among the 162 local rice varieties.

Trait ^a^	SL	SW	ST	BL	BW	BT
SW	−0.158					
ST	0.310	0.736				
BL	0.967	−0.218	0.274			
BW	−0.248	0.971	0.714	−0.281		
BT	0.282	0.705	0.965	0.258	0.698	
100W	0.650	0.531	0.830	0.631	0.491	0.807

^a^ SL: seed length. SW: seed width. ST: seed thickness. BL: brown rice length. BW: brown rice width. BT: brown rice thickness. 100W: 100-seed weight.

**Table 3:** Average values calculated for the seven grain traits among the 162 local rice varieties, based on the allele types of six polymorphic markers. SL: seed length. SW: seed width. ST: seed thickness. BL: brown rice length. BW: brown rice width. BT: brown rice thickness. 100W: 100-seed weight.

Marker	Allele	No.	SL	SW	ST	BL	BW	BT	100W
		acc.	(mm)	(mm)	(mm)	(mm)	(mm)	(mm)	(g)
GW2_SNP	Allele 1	46	9.72	2.72	2.05	6.95	2.28	1.82	2.82
	Allele 2	116	9.16	2.66	2.00	6.58	2.26	1.78	2.53
GS3_SNP1	Allele 1	69	8.57	2.70	1.97	6.10	2.30	1.75	2.31
	Allele 2	93	9.88	2.66	2.04	7.13	2.25	1.82	2.84
GS3_SNP2	Allele 1	95	9.86	2.64	2.03	7.11	2.23	1.81	2.80
	Allele 2	67	8.56	2.74	1.98	6.09	2.33	1.77	2.35
GS5_InDel	Allele 1	112	9.51	2.65	2.02	6.84	2.25	1.80	2.68
	Allele 2	46	8.84	2.74	1.98	6.31	2.32	1.77	2.43
GW5_InDel	Allele 1	42	8.94	2.96	2.09	6.33	2.48	1.87	2.75
	Allele 2	103	9.47	2.53	1.96	6.83	2.15	1.75	2.51
GW6a_InDel2	Allele 1	55	9.64	2.76	2.05	6.92	2.32	1.83	2.83
	Allele 2	107	9.16	2.64	1.99	6.57	2.25	1.78	2.50

**Table 4:** Student’s *t*-test probabilities between the averages of two allele groups of six polymorphic markers for the seven grain traits. SL: seed length. SW: seed width. ST: seed thickness. BL: brown rice length. BW: brown rice width. BT: brown rice thickness. 100W: 100-seed weight.

Marker	SL	SW	ST	BL	BW	BT	100W
GW2_SNP	<0.001 ***	0.215	0.010 *	0.004 **	0.652	0.033 *	<0.001 ***
GS3_SNP1	<0.001 ***	0.472	<0.001 ***	<0.001 ***	0.185	<0.001 ***	<0.001 ***
GS3_SNP2	<0.001 ***	0.031 *	0.012 *	<0.001 ***	0.006 **	0.011 *	<0.001 ***
GS5_InDel	<0.001 ***	0.097	0.035 *	<0.001 ***	0.056	0.040 *	0.003 **
GW5_InDel	0.002 **	<0.001 ***	<0.001 ***	<0.001 ***	<0.001 ***	<0.001 ***	0.003 **
GW6a_InDel2	0.001 **	0.011 *	0.001 **	0.003 **	0.059	0.008 **	<0.001 ***

*, **, ***: Significant at 5%, 1%, and 0.1%, respectively.

## Data Availability

Data are contained in the article and Supplementary Materials.

## Supplementary Materials

The following supporting information can be downloaded at: https://www.mdpi.com/article/10.3390/plants12010133/s1, Figure S1: Original seed envelops; Figure S2: Polyacrylamide gel band patterns of rice local varieties; Table S1: Passport data on 162 local rice varieties collected by Prof. Hamada in Indochinese countries; Table S2: Grain traits measured with 162 local rice varieties in Indochinese countries; Table S3: Information on 19 mutations at 14 grain-size loci; Table S4: Information on the primers to detect 19 functional mutations at 14 grain-size loci; Table S5: Genotypes of 19 mutation sites at 14 grain-size loci examined with 162 local rice varieties from Indochinese countries; Table S6: Three grain traits (hull color, pericarp color, and glutinosity) observed in the 162 local rice varieties; Table S7: Primer information for detecting loss-of-function mutations at *Bh4*, *Rc*, and *Waxy*; Table S8: Comparison of the grain traits among five combinations of genotypes at four marker loci, GW2_SNP, GS5_InDel, GW6a_InDel2, and GS3_SNP1.
