# Supplementary material for: Variations in Grain Traits among Local Rice Varieties Collected More Than Half-Century Ago in Indochinese Countries
Source: Plants (Basel). 2022 Dec 27;12(1):133. doi: 10.3390/plants12010133 (PMC9824235; doi:10.3390/plants12010133)
Supplement: Supplementary file 1 [file plants-12-00133-s001.zip › plants-2058948-supplementary.pdf]

Supplementary Materials

Variations in Grain Traits among Local Rice Varieties Collected More Than Half-Century Ago in Indo–China Countries

Authors:

Sathya Lim, Anna Onoda, Chhourn Orn, Hiromu Iwamoto, Ryo Ishikawa, Hiroki Saito, Yutaka Sato and Takashige Ishii

Affiliated institution (correspondence):

Graduate School of Agricultural Science, Kobe University, 1-1 Rokkodai, Nada-ku, Kobe, Hyogo 657-8501, Japan

Contents

Supplementary Figures S1 – S2

Supplementary Tables S1 – S8

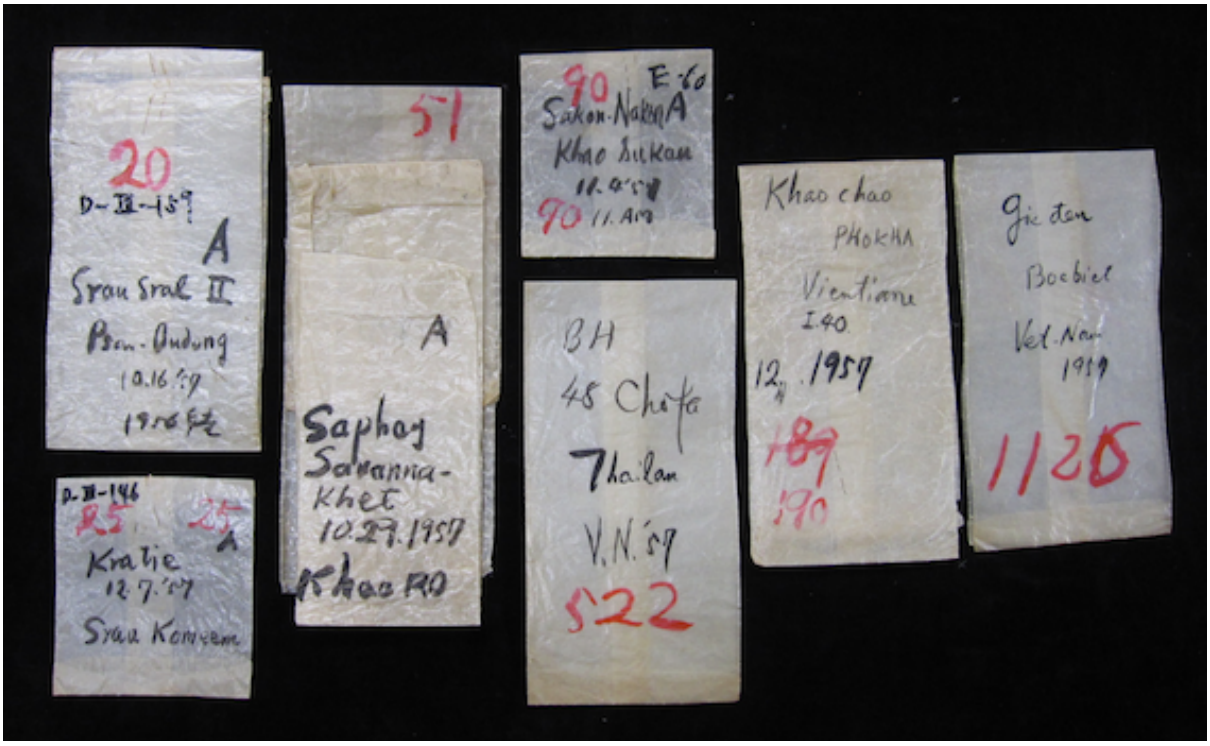

Supplementary Figure S1. Original seed envelops. Prof. Hamada used these envelops to collect seeds of the rice local varieties in Indo–China countries in 1957.

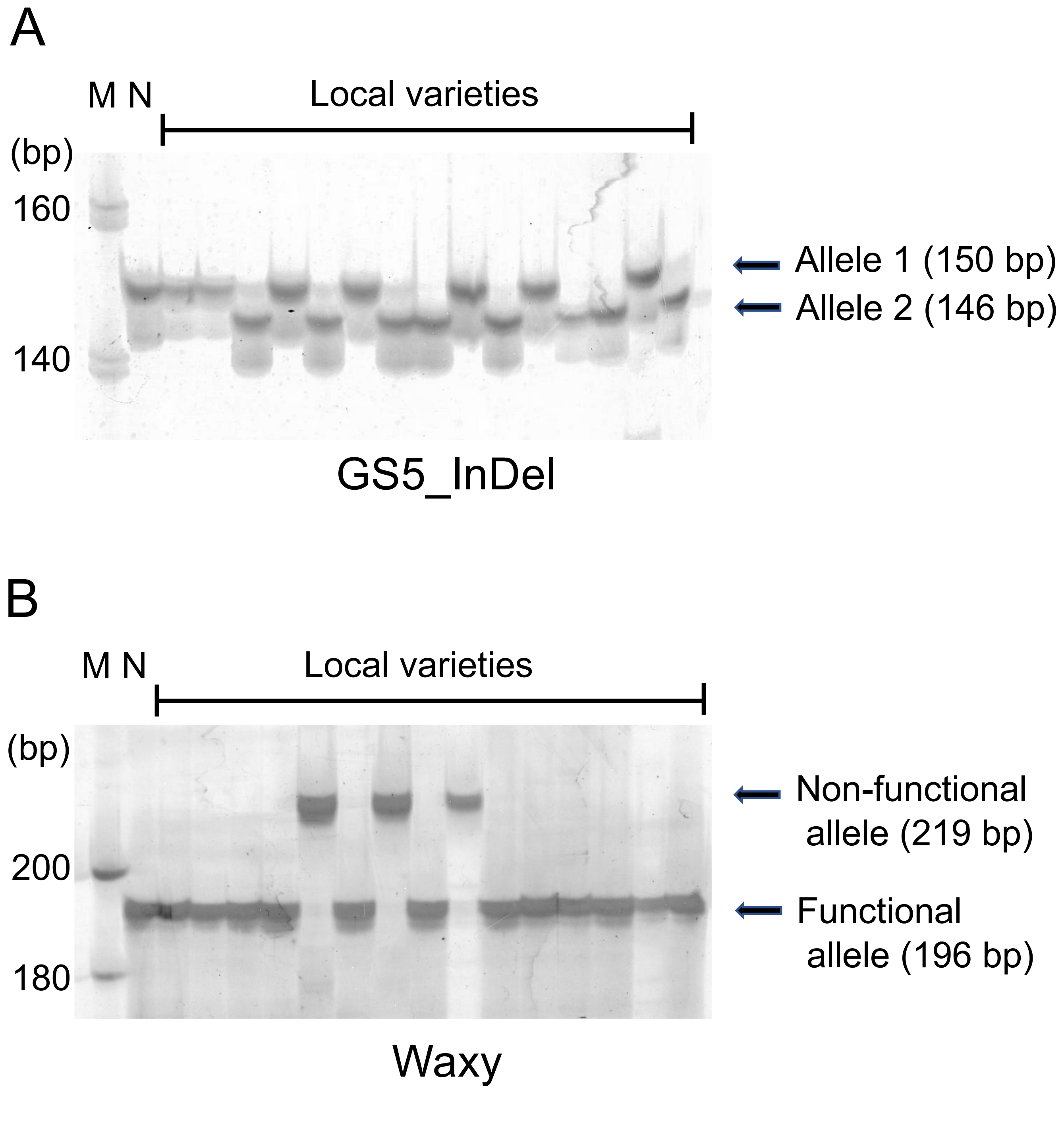

Supplementary Figure S2. Polyacrylamide gel band patterns of rice local varieties. **(A)** GS5_InDel. **(B)** Waxy. M: 20-bp DNA ladder marker. N: Nipponbare. Arrows show the positions of the amplified bands.

Table S1. Passport data on 162 local rice varieties collected by Prof. Hamada in Indo–China countries

Hamada no.[a]	NIG acc. no.[b]	Variety name	Origin	Country	Additional information
20	C6020	Srau Sral II	Psar Oudong	Cambodia	Common nonglutinous rice
25	C6025	Srau Komeem	Kratie	Cambodia	Common nonglutinous rice
51	C6051	Khao Ro	Saphay, Savannakhet	Laos	Low Laos
76	C6076	Khao Pomia	Ban Giyan, Sakon Nakon	Thailand	-
90	C6090	Khao Sukau	Sakon Nakon	Thailand	-
105	C6105	Khao Naman	Ban Phatt, Vientiane	Laos	Central Laos
109	C6109	Unknown	Ban Non Heo, Vientiane	Laos	Central Laos
113	C6113	Khao Puokea	Vientiane	Laos	Central Laos
116	C6116	Khao Deng	Ban Non Heo, Vientiane	Laos	Central Laos
128	C6128	Khao Moe	Ban Tantubou, Vientiane	Laos	Central Laos
143	C6143	Khao Pongeo	Ban Kan, Vientiane	Laos	Central Laos
145	C6145	Khao Lomma	Vientiane	Laos	Central Laos
167	C6167	Khao Khao	Xiengkhouang	Laos	High Laos
170	C6170	Khao Ham	Xiengkhouang	Laos	High Laos
179	C6179	Khao Longma	Vientiane	Laos	Central Laos
186	C6186	Khao Pongseng	Vientiane	Laos	Central Laos
190	C6190	Khao Chao Phokha	Vientiane	Laos	Central Laos
199	C6199	Khao Nio	Ban Sankhalok, Luang Prabang	Laos	High Laos
201	C6201	Khao Cha Han Tat	Ban Pha Teng, Vang Vieng	Laos	High Laos
208	C6208	Khao Chao Noi	Ban Kaison, Vang Vieng	Laos	High Laos
209	C6209	Khao Ipoa	Ban Kaison, Vang Vieng	Laos	High Laos
211	C6211	Khao Nio	Ban Kaison, Vang Vieng	Laos	High Laos
216	C6216	K. Putou	Ban Kaison, Vang Vieng	Laos	High Laos
217	C6217	Khao Khaw	Ban Kaison, Vang Vieng	Laos	High Laos
220	C6220	Sam Pong	Battambang	Cambodia	Common nonglutinous rice
222	C6222	Chang Nay Phdao	Battambang	Cambodia	Common nonglutinous rice
234	C6234	Khoe Soth	Battambang	Cambodia	Common nonglutinous rice
237	C6237	Banteas Phlouk	Battambang	Cambodia	Common nonglutinous rice
238	C6238	Phear Cham	Battambang	Cambodia	Common nonglutinous rice
239	C6239	Phear Cham	Battambang	Cambodia	Common nonglutinous rice
250	C6250	Kabin	Battambang	Cambodia	Common nonglutinous rice
252	C6252	Neang Champa	Battambang	Cambodia	Common nonglutinous rice
254	C6254	Neang Leak	Battambang	Cambodia	Common nonglutinous rice
255	C6255	Samsep Teao	Battambang	Cambodia	Common nonglutinous rice
256	C6256	Neang Ourk	Battambang	Cambodia	Common nonglutinous rice
260	C6260	Sugnuon Thong	Battambang	Cambodia	Common nonglutinous rice
265	C6265	Khao Luong	Battambang	Cambodia	Common nonglutinous rice
318	C6318	Chaeung Sromoch	Battambang	Cambodia	Rice in dry region
393	C6393	Poik Kour	Tonle Sap	Cambodia	Floating rice
429	C6429	Kaotot Long	Battambang	Cambodia	Common nonglutinous rice
431	C6431	Nam Dork Hai	Battambang	Cambodia	Common nonglutinous rice
433	C6433	K.S.T. I	Battambang	Cambodia	Common nonglutinous rice
443	C6443	Khao Horoy	Battambang	Cambodia	Common nonglutinous rice
444	C6444	Neang Champa	Battambang	Cambodia	Common nonglutinous rice
446	C6446	Kao Soi No.70	-	Thailand	-
458	C6458	Kao Taou No. 212	-	Thailand	-
463	C6463	Ham No. 48	-	Thailand	-
467	C6467	Hissitout Venaut	-	Thailand	-
468	C6468	Hai Katium (Haidi)	-	Thailand	-
470	C6470	Kao Chu Bai No.91	-	Thailand	-
473	C6473	Kweopa Sak No. 353	-	Thailand	-
475	C6475	Lueng Leng Long No. 192	-	Thailand	-
476	C6476	Kao Chu Bai No.186	-	Thailand	-
477	C6477	Lueug Ou	-	Thailand	-
478	C6478	Kmer Tia No. 119	-	Thailand	-
480	C6480	Khao Pudas	-	Thailand	-
481	C6481	Bang Pra	-	Thailand	-
483	C6483	Khao Tot Long No.277	-	Thailand	-
485	C6485	Khao Kleo No. 338	-	Thailand	-
489	C6489	Champa Kao No. 370	-	Thailand	-
494	C6494	Lueng Long No. 334	-	Thailand	-
496	C6496	Bang Pra No.287	-	Thailand	-
501	C6501	Chek Ruang No. 379	-	Thailand	-
507	C6507	Khao Vang Nui No. 322	-	Thailand	-
514	C6514	Quang Ngeon	-	Thailand	-
518	C6518	Khao Tot Long	-	Thailand	-
521	C6521	Pey Keo	-	Thailand	-
522	C6522	Chofa	-	Thailand	-
527	C6527	Khao Tot Long	-	Thailand	-
528	C6528	Kong Chud No. 357	-	Thailand	-
531	C6531	Champa Tong No. 54	-	Thailand	-
535	C6535	Champada	-	Thailand	-
643	C6643	Snek Chek	-	Cambodia	Common nonglutinous rice
644	C6644	Neang Tar Changvar	Takeo	Cambodia	Common nonglutinous rice
645	C6645	Neang Pov	Beng Phlong	Cambodia	Common nonglutinous rice
646	C6646	Sambok Ang Krang	-	Cambodia	Common nonglutinous rice
650	C6650	Traneup Kandir	-	Cambodia	Common nonglutinous rice
652	C6652	Srau Seng	-	Cambodia	Common nonglutinous rice
653	C6653	Romang Ker	-	Cambodia	Common nonglutinous rice
655	C6655	Traneup Khmau	-	Cambodia	Common nonglutinous rice
659	C6659	Chang Eng Khum	Prey Nop	Cambodia	Common nonglutinous rice
660	C6660	Chmar Reth	Pratheat	Cambodia	Common nonglutinous rice
661	C6661	Neang Veng No. 340	-	Cambodia	Common nonglutinous rice

664	C6664	Krayar	Kampot	Cambodia	Common nonglutinous rice
666	C6666	Angkor Kraham	-	Cambodia	Common nonglutinous rice
672	C6672	Neang Chan Thugnon	Beng Phlong	Cambodia	Common nonglutinous rice
673	C6673	Theasla Sral	Takeo	Cambodia	Common nonglutinous rice
679	C6679	Kong Rua Reth	-	Cambodia	Common nonglutinous rice
683	C6683	Khmang Romean	-	Cambodia	Common nonglutinous rice
685	C6685	Kraya Choeng No. 355	-	Cambodia	Common nonglutinous rice
687	C6687	Kraya Choeng No.105	-	Cambodia	Common nonglutinous rice
690	C6690	Vir Veay	-	Cambodia	Common nonglutinous rice
691	C6691	Rit Ang Krong	-	Cambodia	Common nonglutinous rice
692	C6692	Kong Chek	-	Cambodia	Common nonglutinous rice
693	C6693	Kraya	-	Cambodia	Common nonglutinous rice
694	C6694	Lek Slek	-	Cambodia	Common nonglutinous rice
695	C6695	Angkor Chen	-	Cambodia	Common nonglutinous rice
697	C6697	Traneup Bey Kher	-	Cambodia	Common nonglutinous rice
726	C6726	Tau Huong 16-1409	Carles	Vietnam	South Vietnam, Early rice of single transplanting
756	C6756	Ravang Thom	Cantho	Vietnam	South Vietnam, Common nonglutinous rice of double transplanting
763	C6763	Bong Dua	Cantho	Vietnam	South Vietnam, Early rice of single transplanting
764	C6764	Nang Choc	Cantho	Vietnam	South Vietnam, Season rice of single transplanting
770	C6770	Tam Vuot	Cantho	Vietnam	South Vietnam, -
779	C6779	Nep Sap	Cholon	Vietnam	South Vietnam, Glutinous rice
784	C6784	Nep Sap	Cholon	Vietnam	South Vietnam, Glutinous rice
786	C6786	Nha Sap	Cholon	Vietnam	South Vietnam, Rice raised in the decreasing waterin winter
791	C6791	Doc Phung Lun A	Giadinh (Ho Chí Minh)	Vietnam	South Vietnam, Half-season rice of single transplanting
793	C6793	Nep Mo	Giadinh (Ho Chí Minh)	Vietnam	South Vietnam, Glutinous rice
800	C6800	Bamonh T2	Bienhoa	Vietnam	South Vietnam, Mountain rice
826	C6826	Doc Phung	Bentre	Vietnam	South Vietnam, Half-season rice of single transplanting
827	C6827	Doc Phung	Bentre	Vietnam	South Vietnam, Half-season rice of single transplanting
831	C6831	Nang Quot Hang 28	Bentre	Vietnam	South Vietnam, Early rice of single transplanting
833	C6833	Doc Nhey Hang 38	Bentre	Vietnam	South Vietnam, Half-season rice of single transplanting
835	C6835	Doc Nhen Hang 24	Bentre	Vietnam	South Vietnam, Half-season rice of single transplanting
850	C6850	Nang Phet Dec	Bentre	Vietnam	South Vietnam, Late rice of single transplanting
854	C6854	Lua Voi	Bentre	Vietnam	South Vietnam, Early rice of single transplanting
859	C6859	Cadung Chum	Thu Dau Mot	Vietnam	South Vietnam, Season rice of single transplanting
867	C6867	Lua Voi	Thu Dau Mot	Vietnam	South Vietnam, Early rice of single transplanting
887	C6887	Cadung Chum	Gocong	Vietnam	South Vietnam, Season rice of single transplanting
895	C6895	Cadung Ket Som	Gocong	Vietnam	South Vietnam, Season rice of single transplanting
913	C6913	Tau Ngu	Phan Thiet	Vietnam	South Vietnam, Early rice of single transplanting
924	C6924	Mong Chim	Phan Thiet	Vietnam	South Vietnam, Half-season rice of single transplanting
934	C6934	Cadung Hue Ky	Rachgia	Vietnam	South Vietnam, Season rice of single transplanting
935	C6935	Nep Co T1	Rachgia	Vietnam	South Vietnam, Glutinous rice
937	C6937	Doc Phung B	Rachgia	Vietnam	South Vietnam, Half-season rice of single transplanting
938	C6938	Doc Phung A	Rachgia	Vietnam	South Vietnam, Half-season rice of single transplanting
951	C6951	Bong Mar	Tayninh	Vietnam	South Vietnam, Early rice of single transplanting
953	C6953	Cadung Chum	Tayninh	Vietnam	South Vietnam, Season rice of single transplanting
960	C6960	Cau Hey	Tayninh	Vietnam	South Vietnam, -
964	C6964	Lua Nep	Vinhlong	Vietnam	South Vietnam, Common nonglutinous rice of double transplanting
965	C6965	Nang Xoi	Vinhlong	Vietnam	South Vietnam, Season rice of single transplanting
976	C6976	Ba Chuc	Soctrang	Vietnam	South Vietnam, Common nonglutinous rice of double transplanting
987	C6987	Bau 157	Soctrang	Vietnam	South Vietnam, -
990	C6990	Nang Le Tanup	Soctrang	Vietnam	South Vietnam, Common nonglutinous rice of double transplanting
1000	C7000	Nang Keo	Soctrang	Vietnam	South Vietnam, Season rice of single transplanting
1006	C7006	Nang Het Den	Soctrang	Vietnam	South Vietnam, Season rice of single transplanting
1008	C7008	Nang Ech	Soctrang	Vietnam	South Vietnam, Common nonglutinous rice of double transplanting
1012	C7012	Chanh 162	Sontay	Vietnam	North Vietnam, Fifth month rice
1013	C7013	Bau 122	Sontay	Vietnam	North Vietnam, Fifth month rice
1014	C7014	Cau 169 B	Sontay	Vietnam	North Vietnam, Fifth month rice
1016	C7016	Ngan	Sontay	Vietnam	North Vietnam, Fifth month rice
1020	C7020	Chanh 205	Sontay	Vietnam	North Vietnam, Fifth month rice
1022	C7022	Hop	Sontay	Vietnam	North Vietnam, Tenth month rice
1024	C7024	Tep Trang 176 T2	Sontay	Vietnam	North Vietnam, Fifth month rice
1026	C7026	Chiem Chanh 198 A	Sontay	Vietnam	North Vietnam, Fifth month rice
1034	C7034	Tam Do 1034	Haiduong	Vietnam	North Vietnam, Tenth month rice
1039	C7039	Gie Noi (Sontay)	Haiduong	Vietnam	North Vietnam, Tenth month rice
1042	C7042	Tam Lua (Sontay)	Haiduong	Vietnam	North Vietnam, Tenth month rice
1093	C7093	Nha Tap (Lua Giang)	Longxuyen	Vietnam	South Vietnam, Rice raised in the decreasing waterin winter
1103	C7103	Hop Va	Bacviet	Vietnam	North Vietnam, Tenth month rice
1105	C7105	Tep Saigon 229	Bacviet	Vietnam	North Vietnam, Fifth month rice
1106	C7106	Giong Chim 351	Bacviet	Vietnam	North Vietnam, Tenth month rice
1108	C7108	Tam Den	Bacviet	Vietnam	North Vietnam, Tenth month rice
1115	C7115	Muong Chim	Bacviet	Vietnam	North Vietnam, -
1117	C7117	Giau Trang ND	Bacviet	Vietnam	North Vietnam, Tenth month rice
1126	C7126	Gie Den	Bacviet	Vietnam	North Vietnam, Tenth month rice
1141	C7141	Nang Ket	Tanan	Vietnam	South Vietnam, Season rice of single transplanting
1142	C7142	Doc Sau	Tanan	Vietnam	South Vietnam, Half-season rice of single transplanting
1149	C7149	Lua Uoi	Tanan	Vietnam	South Vietnam, Floating rice
1152	C7152	Nang Quot	Tanan	Vietnam	South Vietnam, Early rice of single transplanting
1179	C7179	Nep Trung Vit	Camau	Vietnam	South Vietnam, Glutinous rice
1194	C7194	Chantas Phlouk	-	Cambodia	Common nonglutinous rice

[a] Accession number by Prof. Hamada

[b] Accession number in National Institute of Genetics.

Table S2. Grain traits measured with 162 local rice varieties in Indo–China countries. SL: seed length, SW: seed width, ST: seed thickness, BL: brown rice length, BW: brown rice width, BT: brown rice thickness, 100W: 100-seed weight.

Hamada no.[a]	NIG acc. no.[b]	SL (mm)	SW (mm)	ST (mm)	BL (mm)	BW (mm)	BT (mm)	100W (g)
20	C6020	8.39	2.75	1.87	6.09	2.34	1.68	2.22
25	C6025	8.75	2.30	1.81	6.35	1.98	1.66	2.11
51	C6051	10.49	2.42	1.99	7.58	2.07	1.79	2.73
76	C6076	9.55	3.25	2.21	6.65	2.66	1.92	3.23
90	C6090	10.15	2.51	1.98	7.47	2.13	1.78	2.78
105	C6105	10.21	3.43	2.29	7.46	2.90	2.04	4.18
109	C6109	8.44	3.28	2.13	5.71	2.62	1.79	2.45
113	C6113	10.15	2.45	2.00	7.49	2.06	1.80	3.02
116	C6116	8.81	2.76	1.96	6.09	2.31	1.75	2.33
128	C6128	9.76	2.62	2.02	6.75	2.22	1.78	2.63
143	C6143	9.75	2.60	1.97	6.76	2.06	1.72	2.68
145	C6145	10.28	3.10	2.10	7.41	2.48	1.88	3.12
167	C6167	8.77	2.84	2.12	6.23	2.42	1.92	2.60
170	C6170	8.77	2.85	2.03	6.01	2.27	1.81	2.33
179	C6179	10.54	3.22	2.09	7.37	2.62	1.89	3.29
186	C6186	9.76	3.12	2.08	6.93	2.54	1.84	2.95
190	C6190	10.53	2.46	2.03	7.74	2.12	1.82	2.94
199	C6199	10.87	3.46	2.25	7.40	2.81	1.99	3.90
201	C6201	8.81	3.36	2.30	6.14	2.80	1.97	3.26
208	C6208	8.91	2.70	1.94	6.39	2.29	1.73	2.37
209	C6209	8.78	3.45	2.25	6.10	2.85	2.00	2.68
211	C6211	10.08	3.19	2.18	6.75	2.56	1.92	3.04
216	C6216	10.15	3.16	2.27	7.17	2.64	2.00	3.51
217	C6217	10.60	2.97	2.14	7.22	2.48	1.91	3.60
220	C6220	10.63	2.56	2.11	7.53	2.12	1.86	2.93
222	C6222	9.56	2.56	1.92	7.12	2.14	1.72	2.49
234	C6234	9.93	2.71	2.11	7.15	2.32	1.87	3.02
237	C6237	9.39	2.57	1.96	6.74	2.20	1.74	2.52
238	C6238	9.69	2.51	2.06	7.23	2.18	1.88	2.83
239	C6239	9.66	2.73	2.05	7.27	2.33	1.87	2.95
250	C6250	9.09	2.59	1.96	6.65	2.15	1.67	2.38
252	C6252	9.55	2.49	1.96	6.88	2.15	1.76	2.68
254	C6254	10.12	2.74	2.14	7.57	2.32	1.91	3.11
255	C6255	10.48	2.78	2.21	7.66	2.35	1.93	3.26
256	C6256	9.51	2.33	1.93	6.83	2.04	1.74	2.28
260	C6260	10.88	2.32	1.87	7.87	1.93	1.68	2.49
265	C6265	10.93	2.20	1.94	7.66	1.93	1.74	2.51
318	C6318	9.57	2.73	1.99	6.64	2.22	1.74	3.10
393	C6393	8.28	2.49	1.91	5.87	2.18	1.72	1.98
429	C6429	9.46	2.54	1.98	6.95	2.18	1.74	2.65
431	C6431	9.32	2.67	2.06	6.90	2.30	1.83	2.75
433	C6433	10.04	2.53	1.97	7.34	2.13	1.74	2.55
443	C6443	10.00	2.40	2.03	6.95	2.03	1.81	2.56
444	C6444	10.60	2.67	2.00	7.81	2.21	1.79	2.78
446	C6446	10.16	2.69	2.13	7.58	2.27	1.89	3.15
458	C6458	10.16	2.59	2.07	7.42	2.18	1.84	3.06
463	C6463	10.42	2.52	1.99	7.67	2.14	1.79	2.84
467	C6467	9.06	2.47	1.89	6.34	2.03	1.67	2.20
468	C6468	10.01	3.03	2.22	7.00	2.48	1.99	3.31
470	C6470	10.90	2.63	2.16	7.86	2.24	1.92	3.37
473	C6473	10.34	2.71	2.07	7.60	2.29	1.86	3.18
475	C6475	10.04	2.66	2.03	7.42	2.23	1.81	2.79
476	C6476	10.20	2.47	2.01	7.36	2.23	1.76	2.74
477	C6477	10.05	2.54	1.96	7.29	2.15	1.75	2.77
478	C6478	9.68	2.53	1.92	7.02	2.09	1.74	2.48
480	C6480	10.05	2.49	1.99	7.20	2.09	1.79	2.71
481	C6481	9.66	2.57	2.03	6.91	2.15	1.86	2.68
483	C6483	10.43	2.34	2.00	7.58	1.97	1.78	2.77
485	C6485	9.78	2.33	1.99	7.28	2.06	1.79	2.58
489	C6489	9.29	2.85	2.08	6.99	2.45	1.92	3.02
494	C6494	10.35	2.49	2.08	7.66	2.13	1.83	2.99
496	C6496	9.42	2.60	2.06	6.87	2.21	1.79	2.70
501	C6501	9.43	2.53	1.94	6.82	2.13	1.81	2.61
507	C6507	8.84	2.54	1.89	6.35	2.15	1.69	2.26
514	C6514	10.50	2.34	1.99	7.84	1.98	1.77	2.73
518	C6518	9.53	2.50	2.02	6.93	2.14	1.79	2.68
521	C6521	10.57	2.54	2.09	7.86	2.18	1.82	3.06
522	C6522	9.84	2.46	2.02	7.14	2.17	1.82	2.66
527	C6527	9.79	2.52	2.06	7.40	2.12	1.82	2.76
528	C6528	10.65	2.89	2.12	7.73	2.41	1.87	3.67
531	C6531	9.84	2.28	1.99	7.10	1.96	1.80	2.50
535	C6535	9.10	2.52	1.90	6.62	2.11	1.69	2.22
643	C6643	10.45	2.58	2.05	7.92	2.23	1.88	3.07
644	C6644	9.57	2.66	1.96	7.01	2.27	1.72	2.64
645	C6645	9.52	2.28	1.92	6.98	2.04	1.73	2.18
646	C6646	7.81	2.94	1.96	5.57	2.54	1.78	2.31
650	C6650	9.89	2.49	2.07	7.33	2.15	1.86	2.79
652	C6652	10.93	2.70	2.14	7.69	2.23	1.88	3.21
653	C6653	8.27	2.46	1.87	5.85	2.13	1.69	1.98
655	C6655	9.49	2.63	2.05	6.38	2.13	1.84	2.55

659	C6659	9.62	2.30	1.94	6.81	2.03	1.72	2.41
660	C6660	9.16	2.30	1.82	5.99	1.92	1.63	1.76
661	C6661	11.52	3.30	2.26	8.27	2.79	2.02	4.40
664	C6664	11.02	2.45	2.05	7.86	2.05	1.86	2.85
666	C6666	8.54	2.76	1.97	5.88	2.25	1.79	2.18
672	C6672	8.37	2.65	1.89	5.94	2.27	1.69	2.18
673	C6673	8.84	2.55	1.89	6.37	2.23	1.69	2.31
679	C6679	8.90	2.40	1.89	6.52	2.07	1.68	2.34
683	C6683	10.09	2.90	2.19	7.46	2.48	1.96	3.23
685	C6685	10.29	3.00	2.18	7.69	2.56	1.96	3.67
687	C6687	10.47	2.58	2.08	7.69	2.21	1.86	3.01
690	C6690	9.24	2.54	1.95	6.71	2.18	1.71	2.45
691	C6691	9.60	2.31	1.85	7.11	1.94	1.59	2.07
692	C6692	10.32	2.53	1.93	7.77	2.13	1.78	2.74
693	C6693	10.70	2.61	2.04	7.60	2.22	1.78	2.89
694	C6694	9.59	2.60	1.99	6.89	2.16	1.74	2.38
695	C6695	8.06	2.86	1.82	5.73	2.43	1.63	2.10
697	C6697	9.92	2.21	1.78	6.86	1.77	1.59	1.95
726	C6726	8.98	2.93	2.10	6.39	2.47	1.85	2.53
756	C6756	9.69	2.32	1.93	6.60	1.98	1.76	2.25
763	C6763	7.49	2.88	2.07	5.42	2.43	1.86	2.28
764	C6764	8.53	2.57	1.96	6.12	2.19	1.70	2.18
770	C6770	8.56	3.08	2.25	5.93	2.57	1.99	2.94
779	C6779	9.83	3.02	2.09	7.02	2.41	1.89	3.06
784	C6784	8.93	2.61	2.04	6.63	2.20	1.82	2.63
786	C6786	8.20	3.17	2.20	5.74	2.72	1.96	2.74
791	C6791	9.87	2.75	2.11	7.09	2.38	1.88	3.16
793	C6793	9.83	2.90	2.03	6.94	2.34	1.79	2.70
800	C6800	8.28	3.07	2.05	5.88	2.64	1.85	2.59
826	C6826	9.75	2.47	1.99	6.75	2.11	1.78	2.52
827	C6827	9.17	2.72	2.11	6.77	2.40	1.85	2.92
831	C6831	9.52	2.44	2.00	7.07	2.09	1.79	2.61
833	C6833	8.91	2.75	2.04	6.35	2.37	1.85	2.75
835	C6835	8.85	2.65	2.11	6.41	2.29	1.89	2.52
850	C6850	8.64	3.25	2.22	6.10	2.79	2.03	3.22
854	C6854	7.24	3.20	2.20	5.17	2.78	2.01	2.57
859	C6859	7.15	2.73	1.91	5.14	2.39	1.67	1.88
867	C6867	8.89	2.42	1.85	6.50	2.05	1.61	2.07
887	C6887	7.44	3.20	2.12	5.41	2.74	1.88	2.57
895	C6895	7.54	3.13	2.07	5.31	2.77	1.88	2.57
913	C6913	8.19	2.23	1.84	5.79	1.96	1.67	1.81
924	C6924	8.62	3.23	2.23	5.87	2.64	1.97	2.81
934	C6934	7.90	2.99	2.07	5.69	2.52	1.85	2.60
935	C6935	10.26	2.75	2.04	6.83	2.20	1.77	2.67
937	C6937	8.28	2.90	1.98	5.98	2.49	1.82	2.39
938	C6938	7.97	2.88	1.90	5.62	2.44	1.65	2.22
951	C6951	9.07	2.38	1.94	6.50	1.98	1.71	2.26
953	C6953	7.87	2.99	2.11	5.57	2.55	1.92	2.77
960	C6960	8.13	3.08	2.13	5.75	2.62	1.92	2.67
964	C6964	9.02	2.51	1.98	6.75	2.16	1.79	2.23
965	C6965	9.25	2.64	2.13	6.67	2.25	1.89	2.64
976	C6976	8.21	2.78	2.05	5.93	2.42	1.86	2.49
987	C6987	7.52	2.78	1.83	5.34	2.37	1.66	2.00
990	C6990	8.99	2.73	2.04	6.42	2.32	1.83	2.73
1000	C7000	8.45	2.56	1.86	6.08	2.19	1.63	2.06
1006	C7006	8.14	3.19	2.21	5.68	2.77	1.99	2.89
1008	C7008	9.11	2.74	2.04	6.51	2.36	1.81	2.95
1012	C7012	9.20	2.40	1.90	6.71	2.12	1.66	2.32
1013	C7013	7.70	2.62	1.95	5.46	2.36	1.72	2.07
1014	C7014	8.13	2.45	1.82	5.83	2.12	1.60	1.90
1016	C7016	9.05	2.43	1.86	6.69	2.06	1.64	2.16
1020	C7020	8.19	2.49	1.79	5.93	2.12	1.58	1.85
1022	C7022	8.44	2.66	1.91	5.89	2.26	1.72	2.08
1024	C7024	8.13	2.34	1.80	5.89	2.05	1.62	1.83
1026	C7026	8.81	2.40	1.89	6.48	2.02	1.69	2.25
1034	C7034	9.14	2.55	1.83	6.24	2.22	1.61	2.06
1039	C7039	8.31	2.34	1.75	5.76	2.02	1.57	1.73
1042	C7042	8.37	2.49	1.77	5.70	2.16	1.54	1.71
1093	C7093	9.13	2.35	1.90	6.74	2.02	1.72	2.21
1103	C7103	8.06	2.49	1.85	5.73	2.17	1.67	1.90
1105	C7105	8.47	2.63	1.87	6.05	2.19	1.67	2.06
1106	C7106	8.21	2.44	1.88	5.92	2.10	1.68	2.03
1108	C7108	8.28	2.60	1.83	5.93	2.25	1.62	1.97
1115	C7115	8.13	2.63	1.82	5.79	2.27	1.63	1.89
1117	C7117	8.67	2.60	1.97	5.88	2.30	1.77	2.29
1126	C7126	8.34	2.98	2.06	6.03	2.49	1.86	1.89
1141	C7141	9.87	2.63	2.03	7.20	2.21	1.80	2.83
1142	C7142	7.86	2.53	1.86	5.60	2.11	1.67	1.95
1149	C7149	8.14	2.94	2.10	5.78	2.56	1.94	2.57
1152	C7152	10.36	2.29	1.92	7.47	1.95	1.70	2.38
1179	C7179	10.00	2.95	2.12	6.91	2.39	1.96	3.18
1194	C7194	10.68	2.47	2.08	7.56	2.07	1.89	2.67

[a] Accession number by Prof. Hamada

[b] Accession number in National Institute of Genetics.

Table S3. Information on 19 mutations at 14 grain-size loci. They were previously referred to by Zhang et al. (2020).

Locus	Gene ID	Type	Region	Position[a]	Change[b]	Effect[c]	Code[d]
GW2	Os02g0244100	SNP	Promoter	-858	T to A	Grain trait variation	GW2_SNP
		InDel	Exon	c.316	1-bp deletion	Increasing grain width	GW2_InDel
GS2	Os02g0701300	SNP	Exon	c.487 ~ 488	TC to AA	Increasing grain size	GS2_SNP
qLGY3	Os03g0215400	InDel	Intoron	g.7961 ~ 7975	2-bp deletion[e]	Increasing grain length	qLGY3_InDel
GS3	Os03g0407400	SNP	Exon	c.165	C to A	Increasing grain length	GS3_SNP1
		SNP	Intoron	g.4893 ~ 4894	TG to CC	-	GS3_SNP2
GL3.1	Os03g0646900	SNP	Exon	c.1092	C to A	Increasing grain length	GL3.1_SNP
TGW3	Os03g0841800	SNP	Exon	c.672	C to A	Increasing grain length	TGW3_SNP
GS5	Os05g0158500	InDel	Promoter	-324 ~ -327	4-bp deletion	Increasing grain size	GS5_InDel
GW5	Os05g0187500	InDel	Promoter	-4810	1212-bp insertion[f]	Increasing grain width	GW5_InDel
GS6	Os06g0127800	InDel	Promoter	-779	12-bp insertion	Grain size	GS6_InDel
TGW6	Os06g0623700	InDel	Exon	c.312	1-bp deletion	Increasing grain weight	TGW6_InDel
		SNP	Exon	c.690	G to T	-	TGW6_SNP
GW6a	Os06g0650300	InDel	Promoter	-584 ~ -589	6-bp deletion	Increasing grain size	GW6a_InDel1
		InDel	Promoter	-1596 ~ -1620	25-bp deletion	Increasing grain size	GW6a_InDel2
GLW7	Os07g0505200	InDel	5'UTR	-135 ~ -140	6-bp deletion	Increasing grain size	GLW7_InDel
GL7	Os07g0603300	InDel	5'UTR	-104 ~ -114	11-bp deletion	Grain trait variation	GL7_InDel1
		InDel	5'UTR	-145 ~ -162	18-bp deletion	Grain trait variation	GL7_InDel2
GW8	Os08g0531600	InDel	5'UTR	-23	10-bp insertion	Increasing grain size	GW8_InDel

[a] Relative base position to the coding start site based on the promoter and UTR sequences (-), CDS (c.), and genomic sequences (g.).

[b] Change from the reference nucleotide sequences (Nipponbare).

[c] Effect (including putative) caused by the mutation.

[d] Mutation code by Zhang et al. (2020).

[e] Balance of 15-bp deletion and 13-bp insertion.

[f] Around 1000 ~ 1200-bp insertion variation was detected in this study.

Table S4. Information on the primers to detect 19 functional mutations at 14 grain-size loci.

Marker[a]	Forward primer[b]	Reverse primer[b]	Marker type	Enzyme[c]	Allele size (bp) [d]	
					Allele 1	Allele 2
GW2_SNP	AAAACCTAACACGTGGATACAAcA	TTTCGTGTTGGTCAGCATCTTT	dCAPS	*Nsp* I (RCATGY)	165	189
GW2_InDel	CCTTGGCAGTTGGCAATCTA	CGATACTCCACAGCATAACTGG	InDel	-	99	98
GS2_SNP	GCACCGCGGCCGCAtaCGT	TGCCATTGGAGGCAGCAGCA	dCAPS	*Sna* BI (TACGTA)	119	102
qLGY3_InDel	TTGGGCCTAATTTTGTCTTTG	GTCCATGTAGGCCTGGTGAT	InDel	-	140	138
GS3_SNP1	CGCTGCCTCCAGATGgcG	AGTTGCTTAAAAAGATAACGGTCAAA	dCAPS	*Hha* I (GCGC)	141	158
GS3_SNP2	ATGAAAACCTTGTCTGCCACA	AACGTGTGATTTAATCGTTAACggC	dCAPS	*Hae* III (GGCC)	151	175
GL3.1_SNP	CGATTCTATCTGGTTCAGTGgTcGA	TTACCTCCTCGTAGACCTCCA	dCAPS	*Sal* I (GTCGAC)	179	202
TGW3_SNP	GGAACATTTATGCAGACAAACTG	CCAAAATCACATATTTTGAGCcG	dCAPS	*Hpa* II (CCGG)	147	169
GS5_InDel	TCCCATGGAATTACTAGAGAAGC	TATCCATCGTCCATGGTGAA	InDel	-	150	146
GW5_InDel	ACTCCGATTGGCATCACTGT	GGAGGAGGACGGAGTGGT	InDel	-	705	(1600-2000)[e]
GS6_InDel	CTTCTTCCCTTCCCTTCCAC	TGCCTCACCATTACCACTCC	InDel	-	112	124
TGW6_InDel	ACACGTACAGCCCCAGCTAC	TTGCCGGTTTTGTAGTGAAA	InDel	-	118	117
TGW6_SNP	ATGCGAGTTGGTCCAAAAGG	GCAACGATCAGATGTGTTCGGTCAGaGCT	dCAPS	*Sac* I (GAGCTC)	269	296
GW6a_InDel1	CTCGTGTTTCTTGTGCATTTG	TTGACTTATCAGCCGCACTG	InDel	-	265	259
GW6a_InDel2	CGTGCTCCGATAAATAGCTAAAA	CGTTGAGTTTGCATGTTCGT	InDel	-	143	118
GLW7_InDel	CCCGCCTTTATATCCCTTTC	CGAGCTCATGAGTGGAAGTG	InDel	-	111	105
GL7_InDel1[f]	TCTCCCTCTCCTCACGACAC	AAGGAGCAAGCAGAGGTACG	InDel	-	134	123
GL7_InDel2[f]	TCTCCCTCTCCTCACGACAC	AAGGAGCAAGCAGAGGTACG	InDel	-	134	116
GW8_InDel	AGCTTACGCGGGAGCTAA	CGCTGTTCTCCAGCTCGT	InDel	-	115	125

[a] Marker is named after mutation code in Table S3.

[b] Small letters in red indicate modified nucleotide sequences for dCAPS marker.

[c] Enzyme used for dCAPS marker. Recognition sequences are shown in parenthesis.

[d] Alleles 1 and 2: Nipponbare and non-Nipponbere types, respectively.

[e] Allele sizes changed because of the insertion variation.

[f] A same primer pair can detect both InDel mutations at *GL7* .

Table S5. Genotypes of 19 mutation sites at 14 grain-size loci examined with 162 local rice varieties from Indo–China countries. 1: Allele 1 (Nipponbare type), 2: Allele 2 (non-Nipponbare type), H: Heterozygous allele.

Hamada no.[a]	NIG acc. no.[b]	*GW2* SNP	*GW2* InDel	*GS2* SNP	*qLGY3* InDel	*GS3* SNP1	*GS3* SNP2	*GL3.1* SNP	*TGW3* SNP	*GS5* InDel	*GW5*[c] InDel	*GS6* InDel	*TGW6* InDel	*TGW6* SNP	*GW6a* InDel1	*GW6a* InDel2	*GLW7* InDel	*GL7* InDel1	*GL7* InDel2	*GW8* InDel
20	C6020	1	1	1	1	1	2	1	1	1	1	2	1	2	2	2	2	1	2	2
25	C6025	1	1	1	1	2	1	1	1	1	2	2	1	2	2	1	2	1	2	2
51	C6051	1	1	1	1	2	1	1	1	1	2	2	1	2	2	1	2	1	2	2
76	C6076	1	1	1	1	2	1	1	1	2	1	2	1	2	2	1	2	1	2	2
90	C6090	1	1	1	1	2	1	1	1	1	2	2	1	2	2	1	2	1	2	2
105	C6105	1	1	1	1	2	1	1	1	1	n.a.	2	1	2	2	1	2	1	2	2
109	C6109	1	1	1	1	1	2	1	1	2	1	2	1	2	2	1	2	1	2	2
113	C6113	2	1	1	1	2	1	1	1	1	2	2	1	2	2	1	2	1	2	2
116	C6116	2	1	1	1	1	2	1	1	2	1	2	1	2	2	1	2	1	2	2
128	C6128	1	1	1	1	2	1	1	1	1	2	2	1	2	2	1	2	1	2	2
143	C6143	1	1	1	1	2	1	1	1	1	2	2	1	2	2	1	2	1	2	2
145	C6145	2	1	1	1	2	1	1	1	2	1	2	1	2	2	1	2	1	2	2
167	C6167	1	1	1	1	1	2	1	1	2	1	2	1	2	2	1	2	1	2	2
170	C6170	1	1	1	1	1	2	1	1	2	1	2	1	2	2	1	2	1	2	2
179	C6179	2	1	1	1	2	1	1	1	2	n.a.	2	1	2	2	1	2	1	2	2
186	C6186	1	1	1	1	2	1	1	1	2	1	2	1	2	2	1	2	1	2	2
190	C6190	2	1	1	1	2	1	1	1	1	2	2	1	2	2	1	2	1	2	2
199	C6199	1	1	1	1	2	1	1	1	2	1	2	1	2	2	1	2	1	2	2
201	C6201	1	1	1	1	1	2	1	1	1	n.a.	2	1	2	2	1	2	1	2	2
208	C6208	2	1	1	1	1	2	1	1	2	1	2	1	2	2	2	2	1	2	2
209	C6209	1	1	1	1	1	2	1	1	1	1	2	1	2	2	1	2	1	2	2
211	C6211	1	1	1	1	2	1	1	1	1	1	2	1	2	2	2	2	1	2	2
216	C6216	1	1	1	1	2	2	1	1	1	1	2	1	2	2	1	2	1	2	2
217	C6217	2	1	1	1	2	1	1	1	1	1	2	1	2	2	2	2	1	2	2
220	C6220	2	1	1	1	2	1	1	1	1	2	2	1	2	2	2	2	1	2	2
222	C6222	2	1	1	1	2	1	1	1	1	2	2	1	2	2	2	2	1	2	2
234	C6234	2	1	1	1	2	1	1	1	1	1	2	1	2	2	2	2	1	2	2
237	C6237	2	1	1	1	1	2	1	1	1	2	2	1	2	2	2	2	1	2	2
238	C6238	2	1	1	1	1	1	1	1	1	n.a.	2	1	2	2	2	2	1	2	2
239	C6239	2	1	1	1	2	1	1	1	1	1	2	1	2	2	2	2	1	2	2
250	C6250	1	1	1	1	2	1	1	1	1	2	2	1	2	2	2	2	1	2	2
252	C6252	2	1	1	1	2	1	1	1	1	2	2	1	2	2	1	2	1	2	2
254	C6254	2	1	1	1	2	1	1	1	1	1	2	1	2	2	2	2	1	2	2
255	C6255	2	1	1	1	2	1	1	1	1	1	2	1	2	2	2	2	1	2	2
256	C6256	2	1	1	1	2	1	1	1	2	2	2	1	2	2	2	2	1	2	2
260	C6260	2	1	1	1	2	1	1	1	1	2	2	1	2	2	2	2	1	2	2
265	C6265	2	1	1	1	2	1	1	1	1	2	2	1	2	2	2	2	1	2	2
318	C6318	1	1	1	1	2	1	1	1	1	2	2	1	2	2	1	2	1	2	2
393	C6393	2	1	1	1	2	2	1	1	2	2	2	1	2	2	2	2	1	2	2
429	C6429	2	1	1	1	2	1	1	1	1	2	2	1	2	2	1	2	1	2	2
431	C6431	2	1	1	1	2	1	1	1	1	2	2	1	2	2	1	2	1	2	2
433	C6433	2	1	1	1	2	1	1	1	1	2	2	1	2	2	2	2	1	2	2
443	C6443	1	1	1	1	2	1	1	1	1	2	2	1	2	2	2	2	1	2	2
444	C6444	2	1	1	1	2	1	1	1	1	2	2	1	2	2	2	2	1	2	2
446	C6446	2	1	1	1	2	1	1	1	1	2	2	1	2	2	2	2	1	2	2
458	C6458	1	1	1	1	2	1	1	1	1	2	2	1	2	2	2	2	1	2	2
463	C6463	1	1	1	1	1	1	1	1	1	2	2	1	2	2	2	2	1	2	2
467	C6467	2	1	1	1	1	2	1	1	1	2	2	1	2	2	1	2	1	2	2
468	C6468	1	1	1	1	2	2	1	1	H	1	2	1	2	2	1	2	1	2	2
470	C6470	1	1	1	1	2	1	1	1	1	2	2	1	2	2	1	2	1	2	2
473	C6473	2	1	1	1	2	1	1	1	1	2	2	1	2	2	2	2	1	2	2
475	C6475	2	1	1	1	2	1	1	1	1	2	2	1	2	2	1	2	1	2	2
476	C6476	2	1	1	1	2	1	1	1	1	2	2	1	2	2	1	2	1	2	2
477	C6477	1	1	1	1	2	1	1	1	1	2	2	1	2	2	2	2	1	2	2
478	C6478	2	1	1	1	2	1	1	1	2	2	2	1	2	2	1	2	1	2	2
480	C6480	1	1	1	1	2	1	1	1	1	2	2	1	2	2	2	2	1	2	2
481	C6481	1	1	1	1	2	1	1	1	1	2	2	1	2	2	2	2	1	2	2
483	C6483	2	1	1	1	2	1	1	1	1	2	2	1	2	2	2	2	1	2	2
485	C6485	2	1	1	1	2	1	1	1	2	2	2	1	2	2	1	2	1	2	2
489	C6489	2	1	1	1	2	1	1	1	2	2	2	1	2	2	2	2	1	2	2
494	C6494	2	1	1	1	2	1	1	1	2	2	2	1	2	2	1	2	1	2	2
496	C6496	2	1	1	1	2	1	1	1	1	2	2	1	2	2	1	2	1	2	2
501	C6501	2	1	1	1	1	1	1	1	1	2	2	1	2	2	2	2	1	2	2
507	C6507	1	1	1	1	1	2	1	1	1	2	2	1	2	2	1	2	1	2	2
514	C6514	1	1	1	1	2	1	1	1	1	2	2	1	2	2	1	2	1	2	2
518	C6518	2	1	1	1	2	1	1	1	1	2	2	1	2	2	1	2	1	2	2
521	C6521	2	1	1	1	2	1	1	1	1	2	2	1	2	2	2	2	1	2	2
522	C6522	2	1	1	1	2	1	1	1	1	2	2	1	2	2	1	2	1	2	2
527	C6527	1	1	1	1	2	1	1	1	1	2	2	1	2	2	2	2	1	2	2
528	C6528	2	1	1	1	2	1	1	1	2	2	2	1	2	2	2	2	1	2	2
531	C6531	1	1	1	1	2	1	1	1	1	2	2	1	2	2	2	2	1	2	2
535	C6535	2	1	1	1	2	1	1	1	1	2	2	1	2	2	2	2	1	2	2
643	C6643	2	1	1	1	2	1	1	1	1	2	2	1	2	2	2	2	1	2	2
644	C6644	2	1	1	1	2	1	1	1	1	2	2	1	2	2	2	2	1	2	2
645	C6645	2	1	1	1	2	1	1	1	2	2	2	1	2	2	2	2	1	2	2
646	C6646	2	1	1	1	1	2	1	1	2	1	2	1	2	2	2	2	1	2	2
650	C6650	2	1	1	1	2	1	1	1	H	2	2	1	2	2	1	2	1	2	2
652	C6652	1	1	1	1	2	1	1	1	1	2	2	1	2	2	2	2	1	2	2
653	C6653	2	1	1	1	1	2	1	1	2	2	2	1	2	2	2	2	1	2	2
655	C6655	2	1	1	1	2	1	1	1	1	1	2	1	2	2	2	2	1	2	2
659	C6659	2	1	1	1	2	1	1	1	1	2	2	1	2	2	2	2	1	2	2
660	C6660	2	1	1	1	1	1	1	1	1	2	2	1	2	2	2	2	1	2	2
661	C6661	1	1	1	1	2	1	1	1	1	n.a.	2	1	2	2	1	2	1	2	2
664	C6664	1	1	1	1	2	1	1	1	1	2	2	1	2	2	2	2	1	2	2
666	C6666	2	1	1	1	1	1	1	1	1	1	2	1	2	2	2	2	1	2	2

672	C6672	2	1	1	1	1	1	1	1	1	n.a.	2	1	2	2	2	2	1	2	2
673	C6673	2	1	1	1	2	1	1	1	1	2	2	1	2	2	2	2	1	2	2
679	C6679	2	1	1	1	1	2	1	1	1	2	2	1	2	2	1	2	1	2	2
683	C6683	2	1	1	1	2	1	1	1	1	n.a.	2	1	2	2	2	2	1	2	2
685	C6685	1	1	1	1	2	1	1	1	2	1	2	1	2	2	1	2	1	2	2
687	C6687	1	1	1	1	2	1	1	1	1	2	2	1	2	2	2	2	1	2	2
690	C6690	2	1	1	1	2	1	1	1	1	2	2	1	2	2	2	2	1	2	2
691	C6691	2	1	1	1	1	1	1	1	1	n.a.	2	1	H	2	2	2	1	2	2
692	C6692	1	1	1	1	2	1	1	1	1	2	2	1	2	2	1	2	1	2	2
693	C6693	2	1	1	1	2	1	1	1	1	2	2	1	2	2	1	2	1	2	2
694	C6694	2	1	1	1	1	2	1	1	1	2	2	1	2	2	1	2	1	2	2
695	C6695	2	1	1	1	1	2	1	1	2	2	2	1	2	2	2	2	1	2	2
697	C6697	1	1	1	1	2	1	1	1	1	2	2	1	2	2	2	2	1	2	2
726	C6726	2	1	1	1	1	2	1	1	2	2	2	1	2	2	2	2	1	2	2
756	C6756	2	1	1	1	2	1	1	1	1	2	2	1	2	2	2	2	1	2	2
763	C6763	2	1	1	1	1	2	1	1	1	1	2	1	2	2	2	2	1	2	2
764	C6764	2	1	1	1	1	2	1	1	1	2	2	1	2	2	2	2	1	2	2
770	C6770	2	1	1	1	1	2	1	1	1	1	2	1	2	2	2	2	1	2	2
779	C6779	2	1	1	1	2	1	1	1	1	1	2	1	2	2	1	2	1	2	2
784	C6784	2	1	1	1	2	1	1	1	1	2	2	1	2	2	2	2	1	2	2
786	C6786	2	1	1	1	1	2	1	1	1	1	2	1	2	2	2	2	1	2	2
791	C6791	2	1	1	1	2	1	1	1	1	2	2	1	2	2	2	2	1	2	2
793	C6793	2	1	1	1	2	1	1	1	1	n.a.	2	1	2	2	1	2	1	2	2
800	C6800	2	1	1	1	1	2	1	1	1	1	2	1	2	2	1	2	1	2	2
826	C6826	2	1	1	1	1	1	1	1	H	n.a.	2	1	2	2	1	2	1	2	2
827	C6827	2	1	1	1	2	1	1	1	1	2	2	1	2	2	2	2	1	2	2
831	C6831	1	1	1	1	1	1	1	1	1	1	2	1	2	2	1	2	1	2	2
833	C6833	1	1	1	1	1	2	1	1	1	2	2	1	2	2	1	2	1	2	2
835	C6835	1	1	1	1	1	2	1	1	1	2	2	1	2	2	1	2	1	2	2
850	C6850	2	1	1	1	2	1	1	1	2	n.a.	2	1	2	2	2	2	1	2	2
854	C6854	2	1	1	1	1	2	1	1	1	1	2	1	2	2	2	2	1	2	2
859	C6859	2	1	1	1	1	2	1	1	1	1	2	1	2	2	2	2	1	2	2
867	C6867	1	1	1	1	1	2	1	1	2	2	2	1	2	2	1	2	1	2	2
887	C6887	2	1	1	1	2	1	1	1	2	n.a.	2	1	2	2	2	2	1	2	2
895	C6895	2	1	1	1	1	1	1	1	1	1	2	1	2	2	2	2	1	2	2
913	C6913	1	1	1	1	1	1	1	1	2	2	2	1	2	2	2	2	1	2	2
924	C6924	2	1	1	1	1	2	1	1	2	1	2	1	2	2	1	2	1	2	2
934	C6934	2	1	1	1	1	2	1	1	1	1	2	1	2	2	2	2	1	2	2
935	C6935	2	1	1	1	2	1	1	1	1	n.a.	2	1	2	2	2	2	1	2	2
937	C6937	2	1	1	1	2	2	1	1	1	n.a.	2	1	2	2	1	2	1	2	2
938	C6938	2	1	1	1	1	2	1	1	1	2	2	1	2	2	2	2	1	2	2
951	C6951	2	1	1	1	1	2	1	1	1	2	2	1	2	2	2	2	1	2	2
953	C6953	2	1	1	1	1	2	1	1	1	1	2	1	2	2	2	2	1	2	2
960	C6960	2	1	1	1	2	2	1	1	1	2	2	1	2	2	2	2	1	2	2
964	C6964	2	1	1	1	1	2	1	1	1	n.a.	2	1	2	2	2	2	1	2	2
965	C6965	2	1	1	1	2	1	1	1	1	2	2	1	2	2	2	2	1	2	2
976	C6976	2	1	1	1	2	2	1	1	1	n.a.	2	1	2	2	2	2	1	2	2
987	C6987	2	1	1	1	1	2	1	1	2	1	2	1	2	2	2	2	1	2	2
990	C6990	2	1	1	1	1	2	1	1	1	1	2	1	2	2	2	2	1	2	2
1000	C7000	2	1	1	1	1	2	1	1	2	2	2	1	2	2	2	2	1	2	2
1006	C7006	2	1	1	1	1	2	1	1	2	1	2	1	2	2	2	2	1	2	2
1008	C7008	1	1	1	1	1	2	1	1	1	n.a.	2	1	2	2	1	2	1	2	2
1012	C7012	2	1	1	1	1	2	1	1	2	2	2	1	2	2	2	2	1	2	2
1013	C7013	2	1	1	1	1	2	1	1	2	1	2	1	2	2	2	2	1	2	2
1014	C7014	2	1	1	1	1	2	1	1	2	2	2	1	2	2	2	2	1	2	2
1016	C7016	2	1	1	1	1	2	1	1	2	2	2	1	2	2	2	2	1	2	2
1020	C7020	2	1	1	1	1	2	1	1	2	2	2	1	2	2	2	2	1	2	2
1022	C7022	2	1	1	1	1	2	1	1	1	2	2	1	2	2	2	2	1	2	2
1024	C7024	2	1	1	1	1	2	1	1	2	2	2	1	2	2	2	2	1	2	2
1026	C7026	2	1	1	1	1	2	1	1	2	2	2	1	2	2	2	2	1	2	2
1034	C7034	2	1	1	1	1	2	1	1	H	2	2	1	2	2	2	2	2	1	2
1039	C7039	2	1	1	1	1	2	1	1	2	2	2	1	1	2	2	2	1	2	2
1042	C7042	2	1	1	1	1	2	1	1	2	2	2	1	2	2	2	2	2	1	2
1093	C7093	2	1	1	1	2	2	1	1	1	2	2	1	2	2	2	2	1	2	2
1103	C7103	2	1	1	1	1	2	1	1	1	2	2	1	2	2	2	2	1	2	2
1105	C7105	2	1	1	1	1	2	1	1	2	2	2	1	2	2	2	2	1	2	2
1106	C7106	2	1	1	1	1	2	1	1	2	2	2	1	2	2	2	2	1	2	2
1108	C7108	2	1	1	1	1	2	1	1	2	2	2	1	2	2	2	2	1	2	2
1115	C7115	2	1	1	1	1	2	1	1	2	2	2	1	2	2	2	2	2	1	2
1117	C7117	1	1	1	1	1	2	1	1	1	2	2	1	2	2	2	2	1	2	2
1126	C7126	2	1	1	1	1	2	1	1	2	1	2	1	2	2	2	2	1	2	2
1141	C7141	2	1	1	1	2	1	1	1	1	2	2	1	2	2	2	2	1	2	2
1142	C7142	2	1	1	1	1	2	1	1	1	2	2	1	2	2	2	2	1	2	2
1149	C7149	2	1	1	1	1	2	1	1	2	1	2	1	2	2	2	2	1	2	2
1152	C7152	1	1	1	1	2	2	1	1	1	2	2	1	2	2	2	2	1	2	2
1179	C7179	2	1	1	1	2	2	1	1	1	1	2	1	2	2	2	2	1	2	2
1194	C7194	2	1	1	1	2	1	1	1	1	2	2	1	2	2	2	2	1	2	2

[a] Accession number by Prof. Hamada

[b] Accession number in National Institute of Genetics.

[c] n.a.: Not available because of no amplification.

Table S6. Three grain traits (hull color, pericarp color, and glutinosity) observed in the 162 local rice varieties. Major natural mutations at *Bh4*, *Rc*, and *Waxy* were also surveyed.

Hamada no.[a]	NIG acc. no.[b]	Hull color	Pericarp color	Glutinosity	*Bh4*[c]	*Rc*[c]	*Waxy*[c]
20	C6020	Straw	White	Non-glutinous	NF	NF	F
25	C6025	Straw	White	Non-glutinous	NF	NF	F
51	C6051	Straw	White	Non-glutinous	NF	NF	F
76	C6076	Straw	White	Glutinous	NF	NF	NF
90	C6090	Straw	White	Non-glutinous	NF	NF	F
105	C6105	Straw	White	Non-glutinous	NF	NF	F
109	C6109	Intermidiate	Purple	Glutinous	NF	NF	NF
113	C6113	Straw	White	Non-glutinous	NF	NF	F
116	C6116	Straw	White	Glutinous	NF	NF	NF
128	C6128	Straw	White	Glutinous	NF	NF	NF
143	C6143	Intermidiate	White	Glutinous	NF	NF	NF
145	C6145	Straw	White	Glutinous	NF	NF	NF
167	C6167	Straw	White	Non-glutinous	NF	NF	F
170	C6170	Intermidiate	Purple	Glutinous	NF	NF	NF
179	C6179	Straw	White	Glutinous	NF	NF	NF
186	C6186	Intermidiate	White	Glutinous	NF	NF	NF
190	C6190	Straw	White	Non-glutinous	NF	NF	F
199	C6199	Intermidiate	White	Glutinous	NF	NF	NF
201	C6201	Straw	White	Glutinous	NF	NF	NF
208	C6208	Straw	White	Non-glutinous	NF	NF	F
209	C6209	Straw	White	Glutinous	NF	NF	NF
211	C6211	Intermidiate	Red	Glutinous	NF	F	NF
216	C6216	Intermidiate	White	Glutinous	NF	NF	NF
217	C6217	Straw	White	Glutinous	NF	NF	NF
220	C6220	Straw	White	Non-glutinous	NF	NF	F
222	C6222	Straw	White	Non-glutinous	NF	NF	F
234	C6234	Straw	White	Non-glutinous	NF	NF	F
237	C6237	Straw	White	Non-glutinous	NF	NF	F
238	C6238	Intermidiate	White	Non-glutinous	NF	NF	F
239	C6239	Intermidiate	White	Non-glutinous	NF	NF	F
250	C6250	Straw	White	Non-glutinous	NF	NF	F
252	C6252	Straw	White	Non-glutinous	NF	NF	F
254	C6254	Intermidiate	White	Non-glutinous	NF	NF	F
255	C6255	Intermidiate	White	Non-glutinous	NF	NF	F
256	C6256	Intermidiate	White	Non-glutinous	NF	NF	F
260	C6260	Straw	White	Non-glutinous	NF	NF	F
265	C6265	Straw	White	Non-glutinous	NF	NF	F
318	C6318	Intermidiate	White	Glutinous	NF	NF	NF
393	C6393	Straw	White	Non-glutinous	NF	NF	F
429	C6429	Straw	White	Non-glutinous	NF	NF	F
431	C6431	Straw	White	Non-glutinous	NF	NF	F
433	C6433	Straw	White	Non-glutinous	NF	NF	F
443	C6443	Straw	White	Non-glutinous	NF	NF	F
444	C6444	Straw	White	Non-glutinous	NF	NF	F
446	C6446	Straw	White	Non-glutinous	NF	NF	F
458	C6458	Straw	White	Non-glutinous	NF	NF	F
463	C6463	Straw	White	Non-glutinous	NF	NF	F
467	C6467	Straw	White	Non-glutinous	NF	NF	F
468	C6468	Intermidiate	White	Glutinous	NF[c]	NF	NF
470	C6470	Straw	White	Non-glutinous	NF	NF	F
473	C6473	Straw	White	Non-glutinous	NF	NF	F
475	C6475	Straw	White	Non-glutinous	NF	NF	F
476	C6476	Straw	White	Non-glutinous	NF	NF	F
477	C6477	Straw	White	Non-glutinous	NF	NF	F
478	C6478	Intermidiate	White	Non-glutinous	NF	NF	F
480	C6480	Straw	White	Non-glutinous	NF	NF	F
481	C6481	Straw	White	Non-glutinous	NF	NF	F
483	C6483	Straw	White	Non-glutinous	NF	NF	F
485	C6485	Straw	White	Non-glutinous	NF	NF	F
489	C6489	Straw	White	Non-glutinous	NF	NF	F
494	C6494	Intermidiate	White	Non-glutinous	NF	NF	F
496	C6496	Straw	White	Non-glutinous	NF	NF	F
501	C6501	Straw	White	Non-glutinous	NF	NF	F
507	C6507	Straw	White	Non-glutinous	NF	NF	F
514	C6514	Straw	White	Non-glutinous	NF	NF	F
518	C6518	Straw	White	Non-glutinous	NF	NF	F
521	C6521	Straw	White	Non-glutinous	NF	NF	F
522	C6522	Straw	White	Non-glutinous	NF	NF	F
527	C6527	Straw	White	Non-glutinous	NF	NF	F
528	C6528	Straw	White	Non-glutinous	NF	NF	F
531	C6531	Straw	White	Non-glutinous	NF	NF	F
535	C6535	Straw	White	Non-glutinous	NF	NF	F
643	C6643	Straw	White	Non-glutinous	NF	NF	F
644	C6644	Straw	White	Non-glutinous	NF	NF	F
645	C6645	Straw	White	Non-glutinous	NF	NF	F
646	C6646	Straw	White	Non-glutinous	NF	NF	F
650	C6650	Straw	White	Non-glutinous	NF	NF	F
652	C6652	Intermidiate	White	Non-glutinous	NF	NF	F
653	C6653	Intermidiate	White	Non-glutinous	NF[c]	NF	F
655	C6655	Intermidiate	Purple	Glutinous	NF	NF	NF

659	C6659	Straw	White	Non-glutinous	NF	NF	F
660	C6660	Straw	White	Non-glutinous	NF	NF	F
661	C6661	Straw	White	Non-glutinous	NF	NF	F
664	C6664	Straw	White	Non-glutinous	NF	NF	F
666	C6666	Straw	White	Glutinous	NF	NF	NF
672	C6672	Straw	White	Non-glutinous	NF	NF	F
673	C6673	Straw	White	Non-glutinous	NF	NF	F
679	C6679	Intermidiate	White	Non-glutinous	NF	NF	F
683	C6683	Intermidiate	White	Non-glutinous	NF	NF	F
685	C6685	Straw	White	Non-glutinous	NF	NF	F
687	C6687	Straw	White	Non-glutinous	NF	NF	F
690	C6690	Straw	White	Non-glutinous	NF	NF	F
691	C6691	Intermidiate	White	Non-glutinous	NF	NF	F
692	C6692	Straw	White	Non-glutinous	NF	NF	F
693	C6693	Straw	White	Non-glutinous	NF	NF	F
694	C6694	Straw	White	Non-glutinous	NF	NF	F
695	C6695	Intermidiate	Red	Non-glutinous	NF	F	F
697	C6697	Intermidiate	White	Glutinous	NF	NF	NF
726	C6726	Straw	White	Non-glutinous	NF	NF	F
756	C6756	Intermidiate	White	Non-glutinous	NF	NF	F
763	C6763	Straw	White	Non-glutinous	NF	NF	F
764	C6764	Straw	White	Non-glutinous	NF	NF	F
770	C6770	Straw	White	Non-glutinous	NF	NF	F
779	C6779	Straw	White	Glutinous	NF	NF	NF
784	C6784	Straw	White	Non-glutinous	NF	NF	F
786	C6786	Straw	White	Non-glutinous	NF	NF	F
791	C6791	Intermidiate	White	Non-glutinous	NF	NF	F
793	C6793	Straw	White	Glutinous	NF	NF	NF
800	C6800	Straw	White	Non-glutinous	NF	NF	F
826	C6826	Intermidiate	White	Non-glutinous	NF	NF	F
827	C6827	Intermidiate	White	Non-glutinous	NF	NF	F
831	C6831	Straw	White	Non-glutinous	NF	NF	F
833	C6833	Straw	White	Non-glutinous	NF	NF	F
835	C6835	Straw	White	Non-glutinous	NF	NF	F
850	C6850	Intermidiate	White	Non-glutinous	NF	NF	F
854	C6854	Straw	White	Non-glutinous	NF	NF	F
859	C6859	Straw	White	Non-glutinous	NF	NF	F
867	C6867	Straw	White	Non-glutinous	NF	NF	F
887	C6887	Straw	White	Non-glutinous	NF	NF	F
895	C6895	Straw	White	Non-glutinous	NF	NF	F
913	C6913	Straw	White	Non-glutinous	NF	NF	F
924	C6924	Intermidiate	White	Glutinous	NF	NF	NF
934	C6934	Straw	White	Non-glutinous	NF	NF	F
935	C6935	Intermidiate	White	Glutinous	NF	NF	NF
937	C6937	Intermidiate	White	Non-glutinous	NF	NF	F
938	C6938	Intermidiate	White	Non-glutinous	NF	NF	F
951	C6951	Straw	White	Non-glutinous	NF	NF	F
953	C6953	Straw	White	Non-glutinous	NF	NF	F
960	C6960	Straw	White	Non-glutinous	NF	NF	F
964	C6964	Straw	White	Non-glutinous	NF	NF	F
965	C6965	Straw	White	Non-glutinous	NF	NF	F
976	C6976	Straw	White	Non-glutinous	NF	NF	F
987	C6987	Straw	White	Non-glutinous	NF	NF	F
990	C6990	Straw	White	Non-glutinous	NF	NF	F
1000	C7000	Intermidiate	White	Non-glutinous	NF	NF	F
1006	C7006	Intermidiate	White	Non-glutinous	NF	NF	F
1008	C7008	Straw	White	Non-glutinous	NF	NF	F
1012	C7012	Straw	White	Non-glutinous	NF	NF	F
1013	C7013	Straw	White	Non-glutinous	NF	NF	F
1014	C7014	Straw	White	Non-glutinous	NF	NF	F
1016	C7016	Straw	White	Non-glutinous	NF	NF	F
1020	C7020	Straw	White	Non-glutinous	NF	NF	F
1022	C7022	Straw	White	Non-glutinous	NF	NF	F
1024	C7024	Straw	White	Non-glutinous	NF	NF	F
1026	C7026	Straw	White	Non-glutinous	NF	NF	F
1034	C7034	Intermidiate	White	Non-glutinous	NF	NF	F
1039	C7039	Intermidiate	White	Non-glutinous	NF	NF	F
1042	C7042	Intermidiate	White	Non-glutinous	NF	NF	F
1093	C7093	Straw	White	Non-glutinous	NF	NF	F
1103	C7103	Straw	White	Non-glutinous	NF	NF	F
1105	C7105	Straw	White	Non-glutinous	NF	NF	F
1106	C7106	Straw	White	Non-glutinous	NF	NF	F
1108	C7108	Intermidiate	White	Non-glutinous	NF	NF	F
1115	C7115	Straw	White	Non-glutinous	NF	NF	F
1117	C7117	Intermidiate	White	Non-glutinous	NF	NF	F
1126	C7126	Intermidiate	Red	Non-glutinous	NF	F	F
1141	C7141	Straw	White	Non-glutinous	NF	NF	F
1142	C7142	Intermidiate	White	Non-glutinous	NF	NF	F
1149	C7149	Intermidiate	White	Non-glutinous	NF	NF	F
1152	C7152	Straw	White	Non-glutinous	NF	NF	F
1179	C7179	Straw	White	Glutinous	NF	NF	NF
1194	C7194	Straw	White	Non-glutinous	NF	NF	F

[a] Accession number by Prof. Hamada

[b] Accession number in National Institute of Genetics.

[c] F: Functional allele. NF: Non-functional allele.

Table S7. Primer information for detecting loss-of-function mutations at *Bh4* , *Rc* , and *Waxy* .

Locus	Forward primer (5'-3')	Reverse primer (5'-3')	Mutation	Allele size	
				Functional	Non-functional
Bh4	CAACCAGATGCTAGTGATATGC	AGGTTGAGCGTCACCTG	22-bp deletion	114 bp	92 bp
Rc	CAGAAACACCTGAATCAAGGG	CTCTTTCAGCACATGGTTG	14-bp deletion	98 bp	84 bp
Waxy	TGCAGAGATCTTCCACAGCA	GCTGGTCGTCACGCTGAG	23-bp insertion	196 bp	219 bp

Table S8. Comparison of the grain traits among five combinations of genotypes at four marker loci, GW2_SNP, GS5_InDel, GW6a_InDel2, and GS3_SNP1. SL: seed length, ST: seed thickness, BL: brown rice length, BT: brown rice thickness, and 100W: 100-seed weight. The values are averages ± S.D.

Genotype[a]				No. varieties	SL (mm)	SW[b] (mm)	ST[b] (mm)	BL (mm)	BW[b] (mm)	BT[b] (mm)	100W[b] (g)
GW2_SNP	GS5_InDel	GW6a_InDel2	GS3_SNP1								
1	1	1	2	12	10.17 ± 0.69	2.71 ± 0.38	2.05 ± 0.15	7.32 ± 0.59	2.28 ± 0.32	1.83 ± 0.13	3.08 ± 0.67
2	1	1	2	14	9.76 ± 0.60	2.62 ± 0.19	2.02 ± 0.04	7.09 ± 0.43	2.23 ± 0.12	1.80 ± 0.04	2.76 ± 0.17
1	2	1	2	4	10.12 ± 0.59	3.21 ± 0.20	2.18 ± 0.07	7.17 ± 0.47	2.64 ± 0.12	1.93 ± 0.06	3.44 ± 0.43
1	1	2	2	14	10.10 ± 0.49	2.11 ± 0.18	2.02 ± 0.10	7.23 ± 0.38	1.80 ± 0.09	1.80 ± 0.09	2.70 ± 0.34
1	1	1	1	7	8.97 ± 0.27	2.85 ± 0.40	2.09 ± 0.14	6.42 ± 0.32	2.42 ± 0.30	1.86 ± 0.11	2.72 ± 0.32
All[c]				162	9.32 ± 0.34	2.68 ± 0.29	2.01 ± 0.12	6.69 ± 0.73	2.27 ± 0.23	1.79 ± 0.11	2.61 ± 0.48

[a] 1: Allele 1 (Nipponbare type), 2: Allele 2 (non-Nipponbare type). Cells of alleles with increasing effect of trait values are colored in pink.

[b] Cells of higher average values than "1112"are colored in yellow.

[c] Overall with 162 local varieties.
